# SERINC5 Mediates a Postintegration Block to HIV-1 Gene Expression in Macrophages

**DOI:** 10.1128/mbio.00166-23

**Published:** 2023-03-28

**Authors:** Pavitra Ramdas, Ajit Chande

**Affiliations:** a Molecular Virology Laboratory, Department of Biological Sciences, Indian Institute of Science Education and Research, Bhopal, Madhya Pradesh, India; Dana-Farber Cancer Institute

**Keywords:** contextualized protein-protein interactions, SERINC5, macrophages, postentry block to viral protein synthesis, protein interactome remodeling, viral RNA capping

## Abstract

HIV-1 antagonizes SERINC5 by redundant mechanisms, primarily through Nef and additionally via envelope glycoprotein. Paradoxically, HIV-1 preserves Nef function to ensure the exclusion of SERINC5 from virion incorporation regardless of the availability of envelope that can confer resistance, suggesting additional roles of the virion-incorporated host factor. Here, we report an unusual mode of SERINC5 action in inhibiting viral gene expression. This inhibition is observed only in the myeloid lineage cells but not in the cells of epithelial or lymphoid origin. We found that SERINC5-bearing viruses induce the expression of RPL35 and DRAP1 in macrophages, and these host proteins intercept HIV-1 Tat from binding to and recruiting a mammalian capping enzyme (MCE1) to the HIV-1 transcriptional complex. As a result, uncapped viral transcripts are synthesized, leading to the inhibition of viral protein synthesis and subsequent progeny virion biogenesis. Cell-type-specific inhibition of HIV-1 gene expression thus exemplifies a novel antiviral function of virion-incorporated SERINC5.

## INTRODUCTION

SERINC5 (serine incorporator 5) is a constitutively expressed multipass membrane protein that was initially identified for its role in incorporating serine into membranes and facilitating the synthesis of serine-derived lipids (1). However, the function of channeling serine into lipid biosynthesis by SERINC5 has been shown to be dispensable for HIV-1 restriction (2, 3). During the biogenesis, SERINC5 is efficiently incorporated into budding HIV-1 virions in the absence of Nef and affects an early step of the viral replication in susceptible target cells (4, 5). The spectrum of SERINC5 activity on virion infectivity appears to be broad and is now also reported against enveloped viruses like severe acute respiratory syndrome coronavirus 2 (SARS CoV-2), hepatitis B virus (HBV), classical swine fever virus (CSFV), and influenza (6–9). Recently, the antiviral function was also linked with its ability to modulate the expression of the proinflammatory cytokines (10) and potentiate type I IFN signaling (11). There has been a consensus that SERINC5 targets early steps of the Nef-defective HIV-1 life cycle (12, 13), with envelope glycoprotein emerging as an additional determinant (14–16), particularly with the open envelope conformation being necessary for antiviral action. Accordingly, it has been suggested that SERINC5 alters the conformation of sensitive envelopes in HIV-1 particles in order to functionally inactivate them (12, 13). While HIV-1 envelopes from primary isolates like ZM109F, ZM249M, and HXB2 are sensitive to SERINC5 restriction, the macrophage-tropic envelopes JRFL, AD8, and YU2 appears relatively resistant (4, 14). Furthermore, variable sensitivity to SERINC5 restriction has also been demonstrated for envelope glycoproteins from other viruses regardless of the mode of entry or receptor tropism. For instance, the envelopes of influenza A virus, amphotropic murine leukemia virus (MLV), and SARS CoV-2 are sensitive to SERINC5, while the envelope glycoproteins of vesicular stomatitis virus (VSV), Ebola, and SARS CoV-1 are resistant to SERINC5 action (4, 16).

Even with the subsistence of a mechanism for envelope-dependent modulation, HIV-1 Nef and Nef-like factors from other viruses, namely, MLV GlycoGag (4), EIAV S2 (17), FeLV GlycoGag (18), SARS CoV-2 ORF7A (6), remarkably have a preserved attribute to downregulate SERINC5 from the plasma membrane. Our discovery of SERINC2 from coelacanths as an antiretroviral factor antagonized by envelope glycoprotein (19) further suggests that envelope-mediated resistance is an ancient mechanism of SERINC counteraction. Despite an envelope-dependent rescue mechanism, the parallel emergence of Nef and Nef-like factors implies that virion-associated SERINC5 functions beyond targeting early steps of the viral life cycle and manifests alternate modes of antiviral action. Here, we report that virion-incorporated SERINC5 mediates a postintegration block to HIV-1 gene expression in target macrophages by promoting the synthesis of uncapped viral transcripts.

## RESULTS

### Envelope sensitivity to SERINC5 restriction varies with cell type.

**FIG 1:**
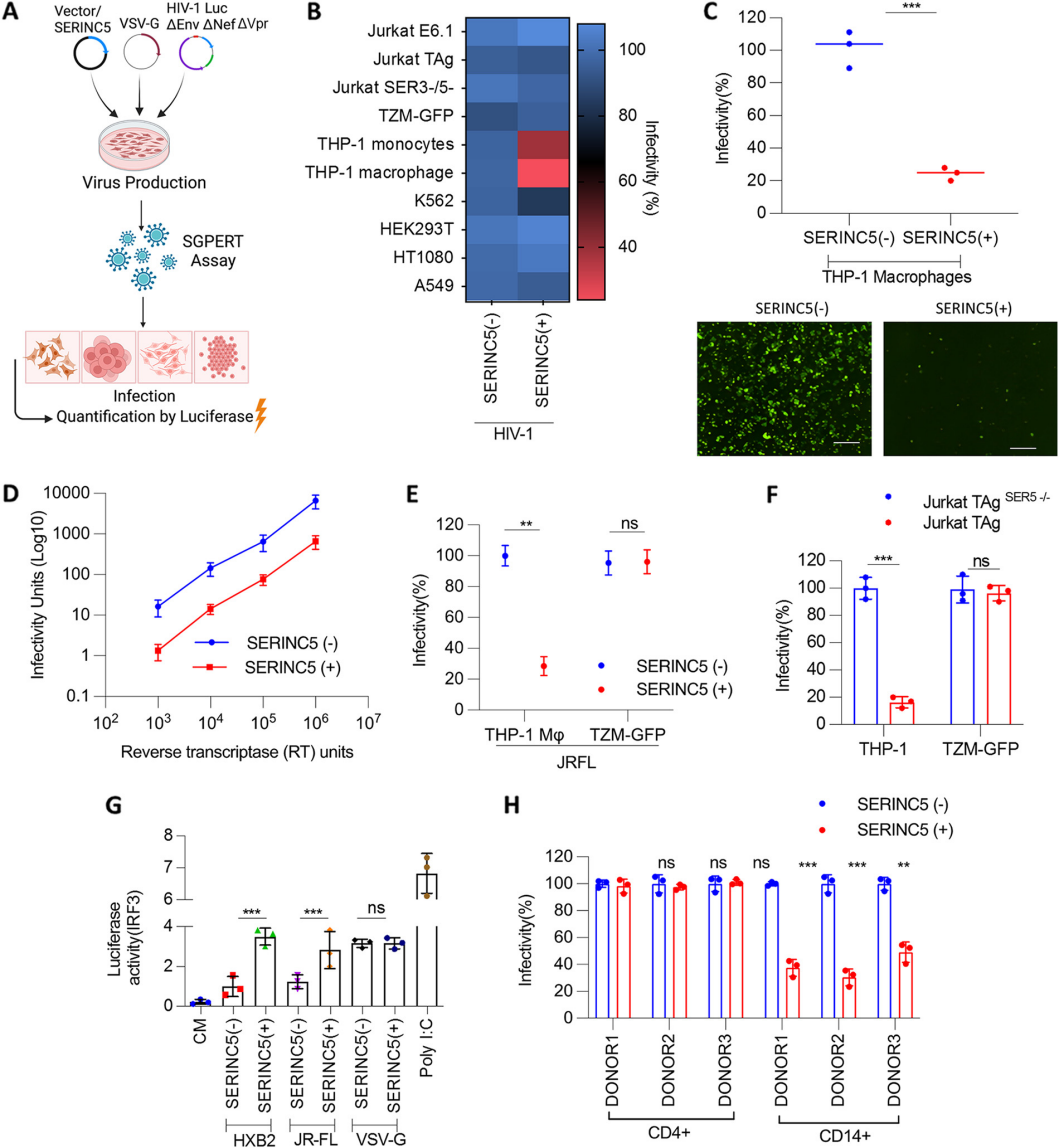
Target cell-type specific infectivity inhibition by SERINC5. (A) Overview of the experimental setup. (B) Indicated cell lines were infected with Nef-defective HIV-1 produced by HEK293T by cotransfecting VSV-G, NL4-3, Luciferase Env(−) R(−), SERINC5, or the equivalent empty vector. Infectivity was normalized to reverse transcriptase (RT) units. The values are means (*n* = 3) and SD. (C) THP-1-derived macrophages were infected with Nef-defective HIV-1 (zsgreen reporter) as in panel B. Infectivity was normalized to RT units. The values are means (*n* = 3) and SD. An unpaired *t* test was used. Lower panels, representative infectivity images from the upper panel. Bar, 200 μm. (D) THP-1-derived macrophages were infected with Nef-defective HIV-1 with increasing amounts of RT units. The values are means (*n* = 3) and SD. (E) THP-1-derived macrophages and TZM-GFP reporter cells were infected with Nef-defective HIV-1 produced in HEK293T by cotransfecting JR-FL env, NL4-3, Luciferase Env(−) R(−), SERINC5, or the equivalent empty vector. Infectivity was normalized to RT units. The values are means (*n* = 3) and SD. An unpaired *t* test was used. (F) THP-1-derived macrophages and TZM-GFP reporter cells were infected with Nef-defective HIV-1 produced by Jurkat TAg and Jurkat TAg ^SER5−/−^ by cotransfecting VSV-G env and NL4-3, Luciferase Env(−) R(−). Infectivity was normalized to RT units. The values are means (*n* = 3) and SD. An unpaired *t* test was used. (G) Quantification of IRF3 activation (Luciferase activity) from the supernatant of THP-1 Dual cells upon challenge with RT-normalized HIV-1 (SERINC5[−] and SERINC5[+]) bearing HXB2, JRFL, or VSV-G envelopes. The values are means (*n* = 3) and SD. An unpaired *t* test was used. (H) Primary CD4^+^ T cells and CD14^+^-derived macrophages obtained from three different healthy donors, enriched to purity (Fig. S7), were infected as in panel B. The values are means (*n* = 3) and SD. An unpaired *t* test was used. *, *P* < 0.05; **, *P* < 0.01; ***, *P* < 0.001; ****, *P* < 0.0001; ns, not significant.

During *in vivo* infection, HIV-1 virions encounter a range of cell types apart from the target lymphoid and myeloid cells. For instance, mucosal epithelial cells are the first sites of contact where virions are internalized and transmigrated to susceptible cells (20). These cells then create an antiviral environment. Meanwhile, the virions escaping from the epithelia gain access to stromal fibroblasts that favor infection and significantly enhance transinfection to T cells (21). Furthermore, considering the extended involvement of the SERINC5 in inhibiting other viruses (SARS CoV-2, HBV, CSFV, and influenza) with various tissue tropisms (6–9), pursuing an assay to test the effect of the host protein on virion infectivity in different cell types appeared promising. The ability of VSV-G to resist SERINC5 antiviral action was earlier established using TZM-GFP as target cells (HEK293T producer cells) (4, 16). While these assays in TZM cells reliably established the resistance of VSV-G and other HIV-1 envelope glycoproteins to SERINC5, the possibility of host factor altering the sensitivity of envelope glycoproteins in a cell-type-specific manner remained to be investigated. With this notion, we challenged cells of distinct tissue origins with VSV-G bearing HIV-1 Luciferase reporter virus (deficient in *env*, *nef*, and *vpr*) (22, 23) that was produced in the presence of either SERINC5, which is here denoted as “HIV-1 (SERINC5+),” or an equivalent amount of an empty vector, which is here denoted as “HIV-1 (SERINC5−)” (Fig. 1A). Curiously, SERINC5 variably affected the infectivity of progeny virions in a cell-type-dependent manner. While the SERINC5 virion association did not result in infectivity impairment in target lymphoblast cells (Jurkat E6.1, Jurkat TAg, Jurkat TAg^SER3-/5-^, K562), fibroblasts (HT1080), or epithelial/epithelial-like cells (TZM-GFP, A549, HEK293T), a noticeable defect in particle infectivity was observed in target THP-1 monocytes and phorbol 12-myristate 13-acetate (PMA)-differentiated THP-1 macrophages (Fig. 1B; Fig. S1A). Notably, these results complied in conditions under which the HIV-1 envelope (HXB2) remained sensitive to virion-incorporated SERINC5 in TZM-GFP cells but not the VSV-G (Fig. S1B). Our subsequent experiments used PMA-differentiated THP-1 (referred to as THP-1 macrophages) as target cells considering the higher degree of inhibition observed in these cells (~10-fold) compared to their undifferentiated counterparts (~5-fold). To ensure that the phenotype was not due to reporter bias or deficiency of Vpr, we produced Vpr-sufficient HIV-1 carrying a zsgreen reporter and found that the infectivity patterns in THP-1 cells mirrored that of HIV-1 carrying the luciferase reporter (Fig. 1C). Notably, the infectivity inhibition seen is not a particle saturation effect (Fig. 1D). Additionally, during the virus production, SERINC5 was also expressed using promoters of different strengths, and a dose-dependent decrease in infectivity was observed in THP-1 macrophages (Fig. S1C). For the remainder of the study, we used SERINC5 expressed from PBJ5- and PBJ6-based plasmids that represent the host protein’s expression levels comparable to physiological conditions, which the viral antagonists can counteract efficiently (4, 24).

10.1128/mbio.00166-23.1 FIG S1 Infectivity of SERINC5+ HIV-1 in cells of distinct tissue origins. (A) RT-normalized infectivity units produced by Nef-defective, SERINC5+/− HIV-1 upon infection of indicated target cells corresponding to Fig. 1B. (B) RT-normalized infectivity (%) of Nef-defective, SERINC5+/− HIV-1 upon infection of THP-1 macrophages and TZM-GFP reporter cells. (C) RT-normalized infectivity (%) of Nef-defective, SERINC5+/− HIV-1 upon infection of THP-1 macrophages. Viruses were produced from HEK293T by transfecting SERINC5 expressors with different promoter strengths. Right panel, western blot showing SERINC5-HA expression and corresponding β-actin from producer cell lysate (HEK293T) and SERINC5-HA and P24 expression from pelleted virions. The values are means (*n* = 3) and SD. An unpaired *t* test was used. *, *P* < 0.05; **, *P* < 0.01; ***, *P* < 0.001; ns, not significant. Download FIG S1, TIF file, 0.7 MB. Copyright © 2023 Ramdas and Chande. 2023 Ramdas and Chande. https://creativecommons.org/licenses/by/4.0/ This content is distributed under the terms of the Creative Commons Attribution 4.0 International license.

Further, it is not a VSV glycoprotein-specific effect, since the viruses produced with macrophage-tropic HIV-1 envelope glycoprotein JR-FL similarly showed reduced infectivity profiles, altogether indicating a SERINC5-dependent effect (Fig. 1E). Additionally, to rule out the effect on infectivity being due to transgenic expression of SERINC5 in HEK293T cells, viruses were also produced from T cells (Jurkat TAg) that endogenously expresses SERINC5 and the counterpart where the endogenous SERINC5 locus is edited by CRISPR/Cas9. Irrespective of the mode of expression or producer cell type, we found that SERINC5 inhibited the infectivity in THP-1 macrophages (Fig. 1F).

Because macrophages are potent effectors of the immune system in response to pathogenic challenges (25), we conjectured whether a heightened innate immune response was underlying such decreases in infectivity. Considering the involvement of SERINC5 (10, 11), it was relevant to check this possibility. We employed THP-1 dual reporter cells to assess the IRF-3 and NF-κB response to SERINC5+/− virus challenge (Fig. 1G; Fig. S2A). However, SERINC5-associated virions did not alter NF-κB responses compared to SERINC5-free virions (Fig. S2B). While HXB2 envelope-bearing HIV-1 poorly infects macrophages compared to JR-FL or VSV-G envelope-bearing virions, in agreement with previous reports (10), we find an elevated type I interferon response induced by SERINC5 with the native, HXB2 envelope (SERINC5-sensitive), as well as the JR-FL (SERINC5-resistant) envelope (Fig. 1G). On the other hand, although VSV-G pseudotyped particles promoted IRF-3 activation, it was indiscernible in the context of SERINC5 (Fig. 1G). These results led us to investigate the infectivity block by virion-associated SERINC5 with the VSV-G envelope. We also confirmed these findings in physiologically relevant primary CD4^+^ cells and CD14^+^-derived macrophages. Consistent with the infectivity profile obtained from monocytic and T-cell lines, target CD14^+^ macrophages displayed lower infectivity with SERINC5(+) viruses compared to SERINC5(−). In contrast, the target CD4^+^ cells were resistant to SERINC5-mediated suppression in infectivity (Fig. 1H; Fig. S2C).

**FIG 2:**
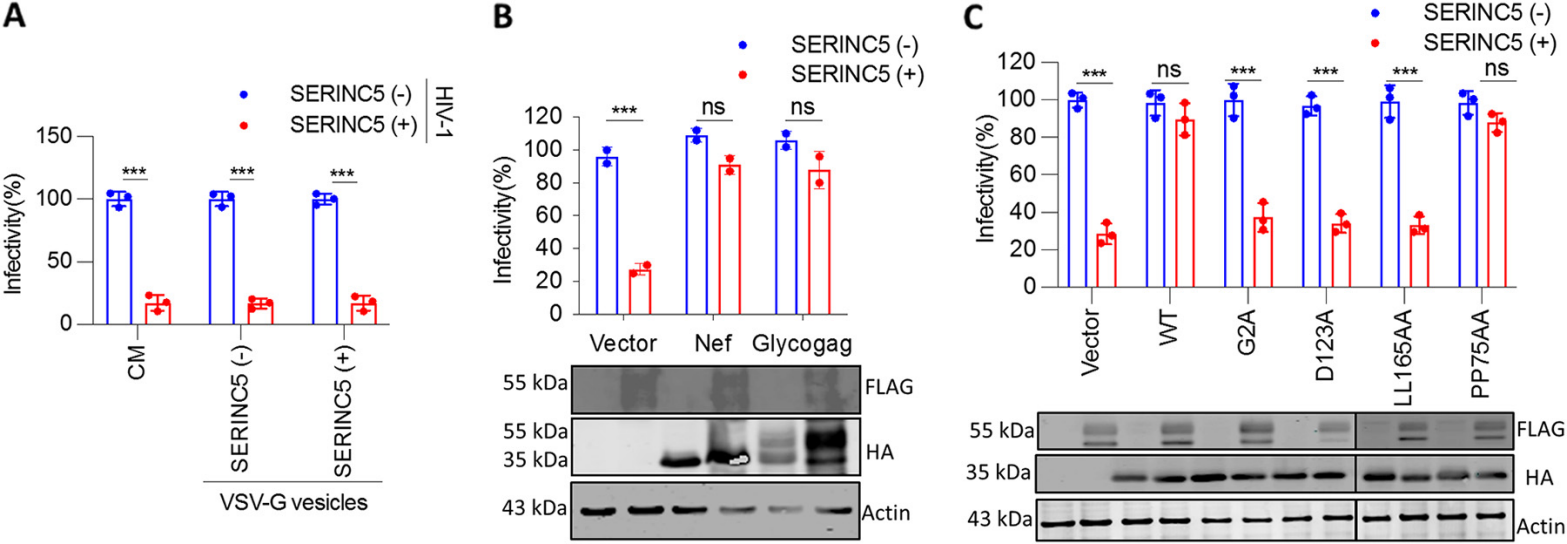
SERINC5 virion association renders the particles defective in THP-1. (A) Infectivity of HIV-1 SERINC5+/− virions in THP-1-derived target macrophages that were primed with either conditioned media (CM) or VSV-G-enveloped vesicles prior to infection with Nef-defective HIV-1. Infectivity was normalized to RT units. Vesicles were produced by transfection of envelope glycoprotein along with empty vector (SERINC5−) or vector expressing SERINC5 (SERINC5+). The values are means (*n* = 3) and SD. An unpaired *t* test was used. (B) THP-1-derived macrophages infected with HIV-1 produced from HEK293T by cotransfecting pMD2.G, NL4-3, Luciferase Env(−) R(−), SERINC5, or equivalent empty vector, along with HIV-1 Nef-HA/MLV-HA-GlycoGag/empty vector. Infectivity was normalized to RT units. Lower panel, Western blot showing C-terminally FLAG-tagged SERINC5 and HA-tagged Nef Lai and GlycoGag with the corresponding β-actin from virus-producing cell lysate (HEK293T). The values are means (*n* = 3) and SD. An unpaired *t* test was used. (C) THP-1-derived macrophages were infected with HIV-1 produced as in panel B with HIV-1 Lai Nef or indicated Nef mutants. Infectivity was normalized to RT units. Lower panel, Western blot showing C-terminally FLAG-tagged SERINC5 and HA-tagged WT and mutants of Nef Lai with the corresponding β-actin from cell lysate (HEK293T). The values are means (*n* = 3) and SD. An unpaired *t* test was used. *, *P* < 0.05; **, *P* < 0.01; ***, *P* < 0.001; ****, *P* < 0.0001; HA, hemagglutinin; MLV, murine leukemia virus; ns, not significant.

10.1128/mbio.00166-23.2 FIG S2 SERINC5-mediated immune induction and infectivity in primary cells and cell lines. (A) Overview of the experimental setup to quantify IRF3 activity and NF-κB activity in THP-1 dual cells. (B) NF-κB response quantification by measuring optical density values at 620 nm (OD_620_) normalized to reverse transcription (RT) from infected THP-1 Dual macrophages. (C) RT-normalized infectivity expressed as infectivity units of Nef-defective, SERINC5+/− HIV-1 in primary cells corresponding to Fig 1H. (D) Western blot showing SERINC5-HA, VSV-G, and CD63 in conditioned medium and VSV-G enveloped vesicles corresponding to Fig. 2A. (E) RT-normalized infectivity (%) of Nef-defective, SERINC5+/− HIV-1 in THP-1, and SERINC5 knockout THP-1 macrophages. (F) RT-normalized infectivity (%) in TZM-GFP reporter cells for Nef-defective HIV-1 complemented with HIV-1 Nef/MLV GlycoGag in the presence and absence of SERINC5. The values are means (*n* = 3) and SD. An unpaired *t* test was used. *, *P* < 0.05; **, *P* < 0.01; ***, *P* < 0.001; MLV, murine leukemia virus; ns, not significant. Download FIG S2, TIF file, 1.3 MB. Copyright © 2023 Ramdas and Chande. 2023 Ramdas and Chande. https://creativecommons.org/licenses/by/4.0/ This content is distributed under the terms of the Creative Commons Attribution 4.0 International license.

### Virion-associated SERINC5 renders the particles defective in THP-1.

We next examined whether SERINC5 must be associated with virions or whether a SERINC5-dependent soluble factor coexpressed regulates the progeny virion inhibition in target macrophages. Toward this, macrophages were primed with VSV-G-enveloped vesicles carrying SERINC5 or conditioned media (detailed in the Materials and Methods section) 6 h prior to infection. The infectivity outcome was insensitive to target cell pretreatment with SERINC5+/− vesicles or CM (Fig. 2A; Fig. S2D), suggesting the host factors’ virion association, and not a coexpressed soluble factor, as a requirement to exert the phenotype.

We also investigated the role of endogenously expressed SERINC5 in target THP-1 macrophages in contributing to the phenotype. Interestingly, it is the SERINC5 association with incoming virions accountable for the observed infectivity defect and not the cell-autonomous expression of the host protein in THP-1 targets cells (Fig. S2E). The gene-edited cells, however, showed an overall increase in infectivity regardless of the presence of SERINC5 in the virions, plausibly due to the attenuation of a SERINC5-dependent immune response (11).

HIV-1 Nef and MLV GlycoGag are known antagonists of SERINC5, which downregulates the host protein from the plasma membrane and excludes it from the progeny virions. In order to examine the effects of viral antagonists of SERINC5 on the reduced infectivity observed in THP-1, we produced HIV-1 (SERINC5+/−) in the presence of Nef and GlycoGag. Expectedly, Nef and GlycoGag expression in the producer cells reversed the phenotype for VSV-G-bearing viruses, reinforcing the requirement of SERINC5 in virus particles for infectivity inhibition THP-1 target cells (Fig. 2B). Of note, the experiments were performed in conditions where Nef and GlycoGag rescued the infectivity inhibition imposed by SERINC5 for HIV-1 particles bearing the native envelope (Fig. S2F), suggesting that neither was the phenotype an artifact of SERINC5 transgenic expression nor was the host factor expression not counteractable by Nef/GlycoGag.

Virion incorporation of SERINC5 is a prerequisite for its antiviral activity, and the viral protein Nef antagonizes the host factors’ antiviral action by counteracting virion-associated pools (26). Nef myristoylation and its interaction with AP2 and dynamin are essential for relocalizing SERINC5 from the plasma membrane to subsequently prevent its incorporation in virions (4). Therefore, in order to finally conclude the requirement of virion-incorporated SERINC5 for the phenotype observed, we employed different Nef mutants that affect Nef myristoylation (G2A), hinder interaction with dynamin 2 (D123A), disrupt AP2 interaction (LL165AA), or interfere with the proline-rich SH3-binding domain (PP75AA). In agreement (4), we find that antagonism-defective Nef mutants failed to rescue the infectivity in THP-1 macrophages (Fig. 2C), thereby confirming that virion-incorporated SERINC5 is mediating the phenotype.

### Virion-incorporated SERINC5 mediates a postintegration block to HIV-1 gene expression.

**FIG 3:**
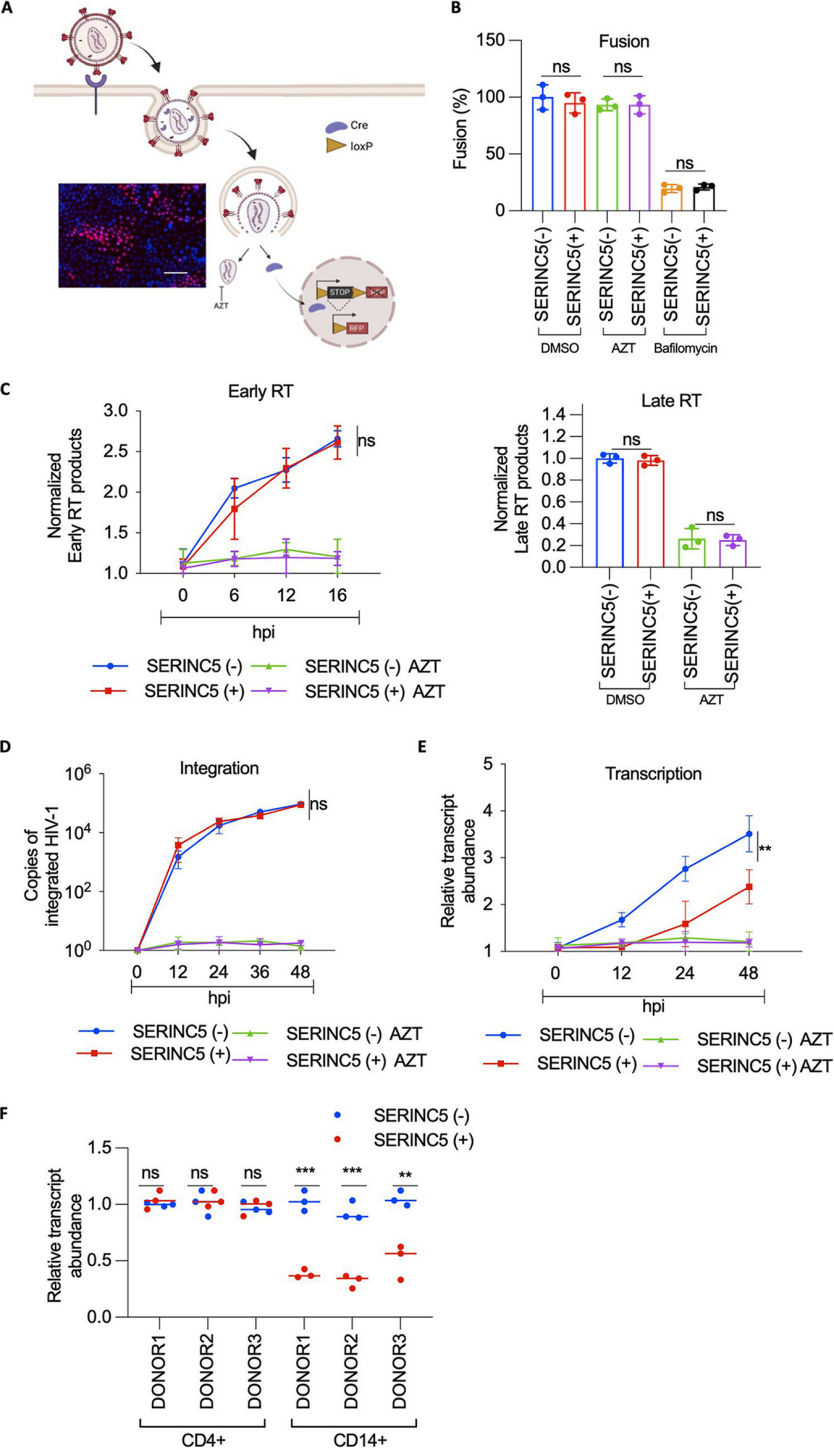
A postintegration block to HIV-1 infectivity in THP-1 macrophages. (A) Representative figure for nlsCre assay to quantify viral fusion. Lower panel, representative image acquired after the assay on the high-content screening (HCS) platform. The nuclei of THP-1 Lox red fluorescent protein (RFP) cells are counterstained with Hoechst, and the RFP signal is visualized in infected cells. Bar, 100 μm. (B) HIV-1 Cre particles produced from HEK293T by cotransfection of VSV-G, 8.9 Cre, SERINC5, or equivalent empty vector were used for scoring fusion efficiency under indicated conditions. Cre-delivery-based fusion was quantified in THP-1 macrophages expressing a Cre-sensitive RFP reporter by scoring the RFP-positive cells. The counts were normalized to RT units. The target cells were treated with either dimethyl sulfoxide (DMSO), zidovudine (AZT), or bafilomycin. The values are means (*n* = 3) and SD. An unpaired *t* test was used. (C) THP-1-derived macrophages were infected with Nef-defective HIV-1 produced from HEK293T by cotransfecting pMD2.G, NL4-3, Luciferase Env(−) R(−), SERINC5, or equivalent empty vector. Early reverse transcription products (early RT) from infected cells (treated with DMSO or AZT) were quantified using quantitative PCR (qPCR) at indicated time points. Lower panel, late reverse transcription products (denoted late RT) from infected THP-1 macrophages (treated with DMSO or AZT) were quantified using qPCR at 24 h postinfection (hpi). The values are means (*n* = 3) and SD. An unpaired *t* test was used. (D) THP-1-derived macrophages infected with Nef-defective HIV-1 produced as in panel B were treated with DMSO or AZT and used to assess proviral integration, quantified using two-step Alu-gag PCR at indicated time points. The values are means (*n* = 3) and SD. An unpaired *t* test was used. (E) THP-1-derived macrophages infected with Nef-defective HIV-1 produced as in panel B were treated with DMSO or AZT and used to assess cytoplasmic viral transcript abundance by qPCR at indicated time points. The values are means (*n* = 3) and SD. An unpaired *t* test was used. (F) The viral transcript abundance in infected CD4^+^ cells and CD14^+^-derived macrophages quantified using qPCR 24 h postinfection. The values are means (*n* = 3) and SD. An unpaired *t* test was used. *, *P* < 0.05; **, *P* < 0.01; ***, *P* < 0.001; ****, *P* < 0.0001; ns, not significant.

SERINC5-mediated particle infectivity inhibition has been directed toward its effect on functional inactivation of envelope leading to nonproductive viral fusion to the target cells. However, considering that the VSV-G envelope is resistant to SERINC5, we anticipated that it would be unlikely for SERINC5 to influence the fusion of VSV-G pseudotyped HIV-1 in macrophages. Nonetheless, we generated a THP-1 cell line (fusion reporter line) with an red fluorescent protein (RFP) reporter, the expression of which is regulated by the Cre recombinase (Fig. 3A). Accordingly, Cre-containing viral particles were produced with VSV-G envelope and SERINC5 or the equivalent empty vector. Upon infection of the fusion reporter line, we find an insignificant difference in the fusion of viral particles (HIV-1 SERINC5+/−). Complying with the assay (detailed in the Materials and Methods section), bafilomycin, but not zidovudine (AZT), can block the activation of fusion reporter (Fig. 3B). Since bafilomycin exposed for an extended time is toxic to cells, the reverse transcription (RT) products were measured to confirm that the drug indeed affected the entry of VSV-G pseudotyped particles (Fig. S3A), suggesting that SERINC5 is not a barrier for the virus entry in this context.

10.1128/mbio.00166-23.3 FIG S3 SERINC5 exerts a postintegration block to HIV-1 gene expression. (A) Early reverse transcription products quantified in the cytoplasmic fraction of infected THP-1 macrophages (treated with DMSO or bafilomycin) using quantitative PCR (qPCR) at the indicated time points. (B) Overview of subcellular fractionation of THP-1 macrophages after infection and fractions taken for assessing various steps of the viral lifecycle. (C) Viral transcription from THP-1 macrophages infected with indicated viruses at different time points quantified using qPCR. (D) Western blot for p24 from infected THP-1 macrophages and corresponding β-actin. The values are means (*n* = 3) and SD. An unpaired *t* test was used. *, *P* < 0.05; **, *P* < 0.01; ***, *P* < 0.001; ns, not significant. Download FIG S3, TIF file, 0.8 MB. Copyright © 2023 Ramdas and Chande. 2023 Ramdas and Chande. https://creativecommons.org/licenses/by/4.0/ This content is distributed under the terms of the Creative Commons Attribution 4.0 International license.

Having learned that the block could be postfusion, we investigated the subsequent steps of the viral life cycle (Fig. S3B). We quantified the early and late RT products but did not observe SERINC5 to have an effect at this stage (Fig. 3C). The proviral DNA integrants were not distinct either (Fig. 3D). Strikingly, the viral transcript levels in the cytoplasm were reduced in the cells infected with HIV-1 (SERINC5+) (Fig. 3E). Furthermore, Nef expression during virus production reversed SERINC5’s effect on viral transcript abundance (Fig. S3C), suggesting that SERINC5 exclusion from virion incorporation by Nef can prevent subsequent blocks to virus gene expression. This difference in transcript abundance was also recapitulated in primary CD14^+^-derived macrophages but not in CD4^+^ cells (Fig. 3F), indicating the cell-specific nature of the block. Together, these results confirmed a postintegration block to HIV gene expression, in which reduced levels of viral RNAs in the cytoplasm were detected.

### Infection from SERINC5+ virions leads to the retention of viral RNA in the nucleus.

**FIG 4:**
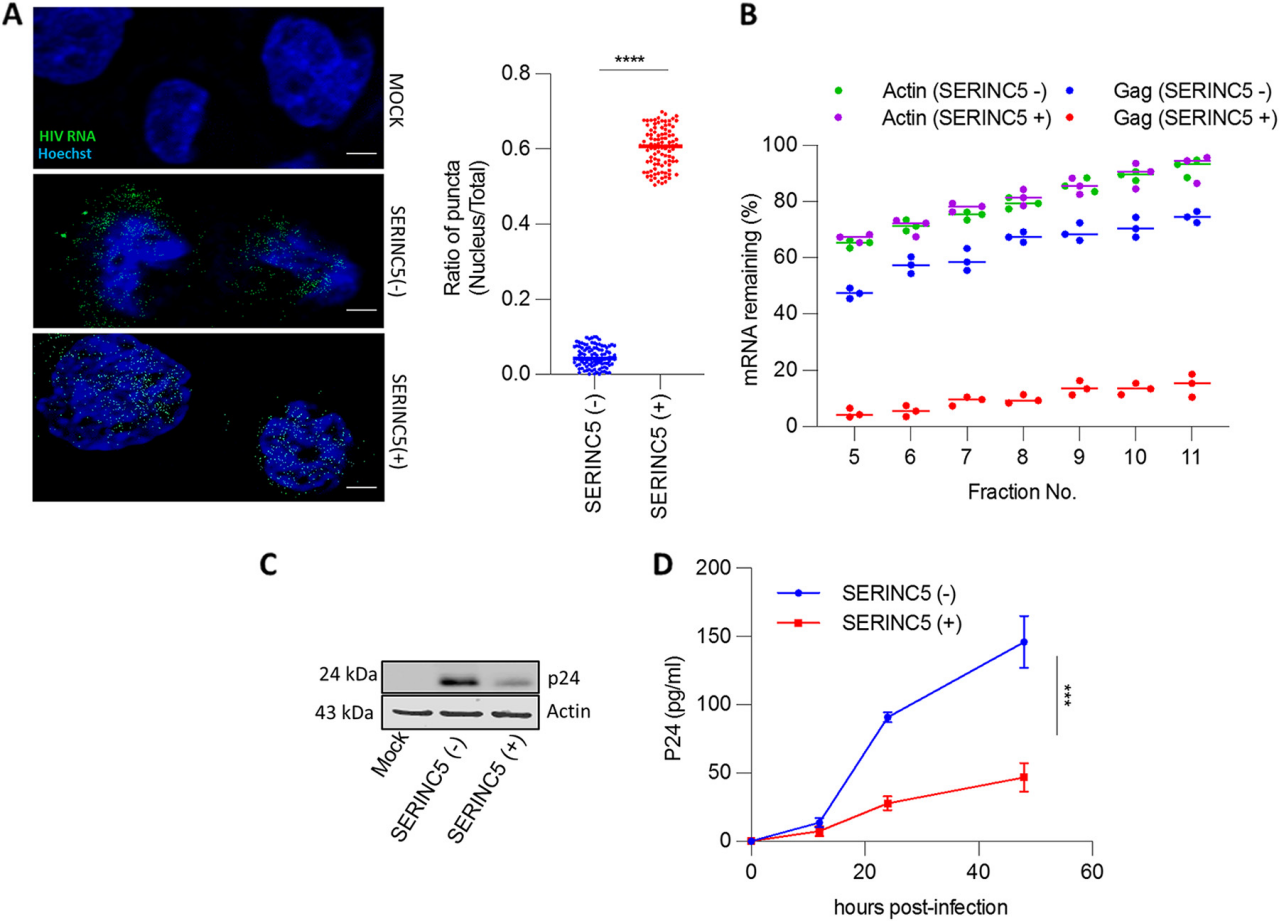
Infection from SERINC5+ virion leads to the retention of viral RNA in the nucleus. (A) HIV-1 RNA was detected by fluorescent *in-situ* hybridization (FISH) 20 h postinfection in THP-1 macrophages infected with SERINC5+/− Nef-defective HIV-1. Green puncta indicate viral RNA, and blue represents the Hoechst-stained nuclei. Bar, 10 μm. Right panel, the ratio of puncta in the nucleus to total puncta in infected cells (*n* = 100). The foci of viral RNA were counted on Fiji-2 software. (B) β-Actin and HIV-1 gag mRNA-remaining (%) quantified by qPCR from the indicated polysome fractions of THP-1-derived macrophages that were infected with SERINC5+/− Nef-defective HIV-1. The values are means (*n* = 3) and SD. An unpaired *t* test was used. (C) Western blot indicating intracellular HIV-1 P24 levels and corresponding β-actin from THP-1-derived macrophages infected with SERINC5+/− Nef-defective HIV-1. (D) Cell-free supernatant from the infected THP-1-derived macrophages collected at the indicated time points and subjected to P24 enzyme-linked immunosorbent assay (ELISA). The values are means (*n* = 3) and SD. An unpaired *t* test was used. *, *P* < 0.05; **, *P* < 0.01; ***, *P* < 0.001; ****, *P* < 0.0001; ns, not significant.

The differential transcript abundance in the subcellular compartments prompted us further to monitor the viral RNA in the infected THP-1 macrophages. Accordingly, we performed RNA fluorescent *in-situ* hybridization (FISH), which revealed that while HIV-1 RNA was efficiently exported to the cytoplasm in the cells infected with HIV-1 (SERINC5−), the viral RNA was predominantly nucleus-localized in the cells infected with HIV-1 (SERINC5+) (Fig. 4A). The retention of viral transcripts within the nucleus in HIV-1 (SERINC5+)-infected cells consequentially hampered its translation, as reflected in the polysome loading of *gag* (Fig. 4B) and the intracellular levels of viral capsid (Fig. 4C). Expectedly, Nef’s presence during virus production could reverse the associated defect in viral protein translation (Fig. S3D). Together, these experiments suggested a postintegration block exerted by virion-incorporated SERINC5, which subsequently leads to impairment of viral RNA transport to the cytoplasm for protein expression. Such a block correspondingly impaired the accumulation of progeny virions in the culture supernatant of infected THP-1 macrophages (Fig. 4D).

### Infection from HIV-1 (SERINC5+) upregulates RPL35 and DRAP1 in macrophages.

**FIG 5:**
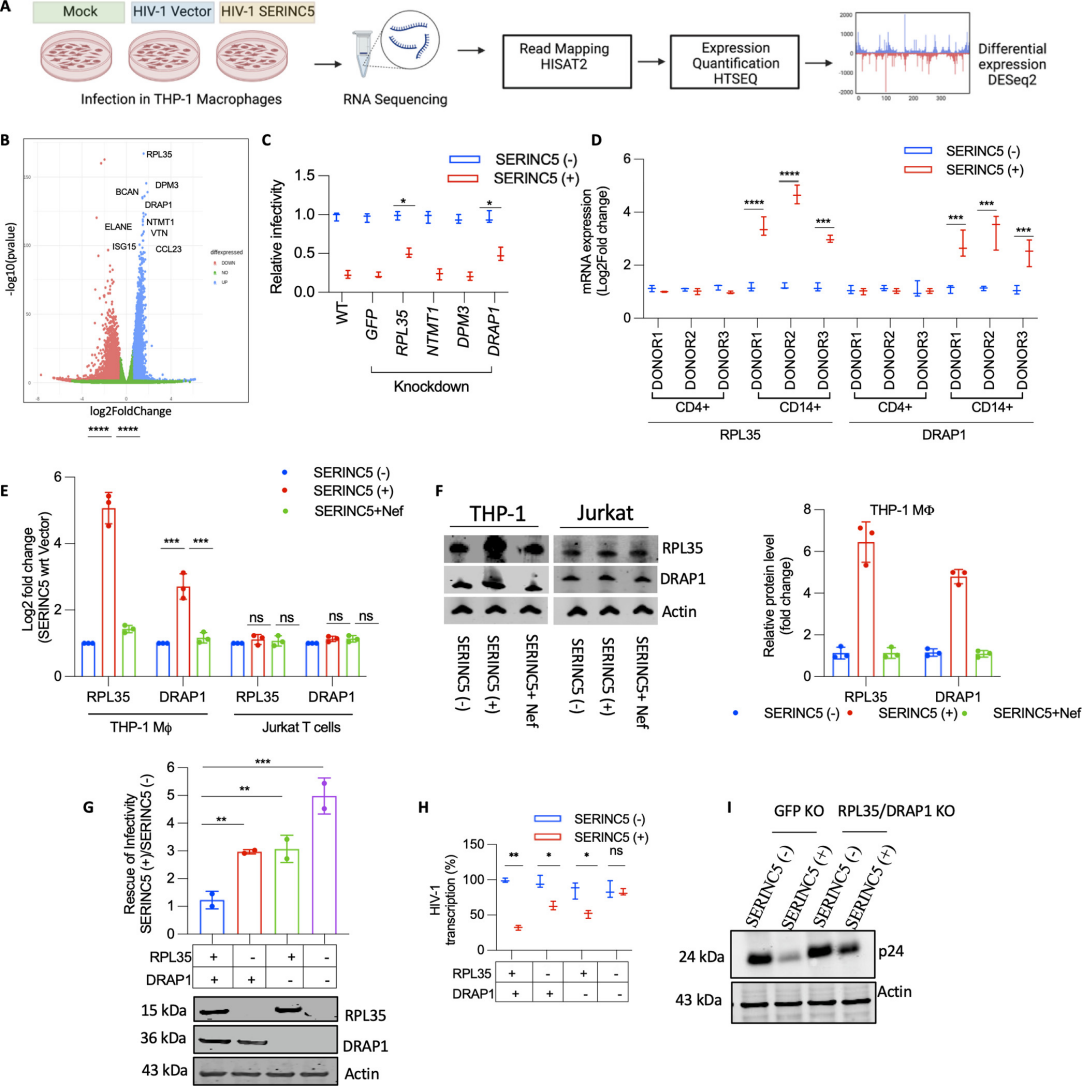
Induction of RPL35 and DRAP1 upon SERINC5 incorporated virion challenge. (A) RNA-sequencing and analysis workflow for scoring differentially expressed genes in the indicated conditions. (B) Differentially expressed genes between HIV-1 (SERINC5−)-infected and HIV-1 (SERINC5+)-infected cells following the RNA-sequencing workflow described in panel A. The values are means (*n* = 2) and SD. (C) RT-normalized infectivity scored from THP-1 target cells after knockdown of the indicated genes by short hairpin RNAs. Nef-defective SERINC5+/− HIV-1 was produced from HEK293T by cotransfecting pMD2G, NL4-3, and Luciferase Env(−), R(−) Nef(−) along with SERINC5 expressor or empty vector. The values are means (*n* = 3) and SD. An unpaired *t* test was used. (D) RPL35 and DRAP1 mRNA expression quantified using qPCR 24 h postinfection from infected CD4^+^ T cells and CD14^+^-derived macrophages from three donors. The values are means (*n* = 3) and SD. An unpaired *t* test was used. (E) RPL35 and DRAP1 mRNA expression quantified using qPCR from THP-1-derived macrophages infected with SERINC5+/SERINC5−/SERINC5+Nef-HIV-1 produced from HEK293T. The values are means (*n* = 3) and SD. An unpaired *t* test was used. (F) Western blotting of RPL35, DRAP1, and the corresponding β-actin from the THP-1 macrophages infected with SERINC5+/SERINC5−/SERINC5+Nef-HIV-1 produced from HEK293T. Right panel, densitometry plot depicting relative RPL35 and DRAP1 protein expression in infected THP-1 macrophages obtained from Western blots (*n* = 3). (G) THP-1 cells lacking RPL35, DRAP1, or RPL35 and DRAP1 were generated. Macrophages derived from these cells were infected with Nef-defective HIV-1 produced as in panel C. The infectivity rescue with SERINC5 in GFP knockout (nonrelevant target) THP-1 macrophages was set to 1 for comparison with the other cell lines. The values are means (*n* = 3) and SD. An unpaired *t* test was used. Lower panel, Western blot showing RPL35, DRAP1 levels and corresponding β-actin in indicated knockout THP-1 cells. (H) THP-1-derived macrophages (GFP, RPL35, and/or DRAP1 knockouts) were infected with Nef-defective HIV-1 produced as in panel C. Viral transcript abundance in the cytoplasm of infected cells was quantified using qPCR 24 h postinfection. The values are means (*n* = 3) and SD. An unpaired *t* test was used. (I) Western blot showing HIV-1 p24 levels and corresponding β-actin from THP-1-derived macrophages infected with Nef-defective HIV-1 produced as in panel C. The values are means (*n* = 3) and SD. An unpaired *t* test was used. *, *P* < 0.05; **, *P* < 0.01; ***, *P* < 0.001; ****, *P* < 0.0001; ns, not significant; WT, wild type.

To find the host cell modifiers leading to the impairment of viral RNA transport, we performed mRNA sequencing of mock-infected or HIV-1 (SERINC5+/−)-infected THP-1 macrophages (Fig. 5A). Differential gene expression analysis (detailed in the Materials and Methods section) revealed candidates that were positively regulated upon infection with HIV-1 (SERINC5+) (Fig. 5B). The transcript levels of the top candidates were also validated by quantitative PCR (qPCR) (Fig. S4A). Those that were upregulated above the threshold were depleted by RNA interference (Fig. S4B), and the infectivity of HIV-1 (SERINC5+/−) was subsequently examined in THP-1 macrophages. We found that the knockdown of RPL35 (ribosomal protein L35) and DRAP1 (DR1-associated corepressor) partially rescued the infectivity inhibition mediated by virion-associated SERINC5 (Fig. 5C). Upregulation of the host genes, RPL35 and DRAP1, were correspondingly also observed in primary HIV-1 (SERINC5+)-infected CD14^+^-derived macrophages but not in CD4^+^ T cells, indicating their role in specific cell types (Fig. 5D). In cell lines, these increases in RPL35 and DRAP1 mRNAs were restricted to THP-1, and Jurkat T cells did not show such profiles (Fig. 5E). Infection from HIV-1 (SERINC5+) in macrophages upregulated RPL35 by ~5-fold and DRAP1 by ~3-fold at the RNA level, which was, importantly, reversed when the viruses were produced in the presence of viral accessary protein Nef (Fig. 5E). Notably, not just the RNA but the corresponding proteins were also upregulated in THP-1 macrophages but not in Jurkat T cells (Fig. 5F).

10.1128/mbio.00166-23.4 FIG S4 Induction of RPL35 and DRAP1 upon SERINC5 incorporated virion challenge. (A) mRNA expression of candidates obtained from RNA-sequencing analysis. (B) mRNA expression of indicated genes quantified by qPCR in various knockdown THP-1 cells generated. (C) RT-normalized infectivity of Nef-defective, SERINC5+/− HIV-1 in WT THP-1 macrophages (lane 1), THP-1^RPL35−^ (lane 2), THP-1^DRAP1−^ (lane 3), THP-1^RPL35−/DRAP1−^ (lane 4), RPL35 re-expressed in THP-1^RPL35−^ (lane 5), DRAP1 re-expressed in THP-1^DRAP1−^ (lane 6), and RPL35 and DRAP1 re-expressed in THP-1^RPL35−/DRAP1−^. Lower panel, Western blot showing RPL35 and DRAP1 levels from infected THP-1 macrophages and corresponding β-actin from infected cell lysate. (D) RT-normalized infectivity of Nef-defective, SERINC5+/− HIV-1 in RPL35 and DRAP1 overexpressed THP-1 macrophages. (E) THP-1 macrophages (GFP, RPL35, and/or DRAP1 Knockouts) were infected with Nef-defective, SERINC5+/− HIV-1. Proviral integration in infected cells was quantified using two-step Alu-gag PCR 24 h postinfection. (F) HIV-1 RNA associated with RPL35 from infected THP-1 macrophages quantified from a PAR-CLIP experiment. (G) HIV-1 long terminal repeat (LTR) enrichment with DRAP1 from infected THP-1 macrophages quantified from a ChIP assay. The values are means (*n* = 3) and SD. An unpaired *t* test was used. *, *P* < 0.05; **, *P* < 0.01; ***, *P* < 0.001; ns, not significant. Download FIG S4, TIF file, 0.9 MB. Copyright © 2023 Ramdas and Chande. 2023 Ramdas and Chande. https://creativecommons.org/licenses/by/4.0/ This content is distributed under the terms of the Creative Commons Attribution 4.0 International license.

To investigate the effect of RPL35 and DRAP1 on HIV-1 infectivity, we next generated knockouts of RPL35 and DRAP1 using CRISPR/Cas9 and examined the infectivity in target THP-1 macrophages. Interestingly, the absence of RPL35 and DRAP1 additively promoted the infectivity rescue (Fig. 5G), and the re-expression restored the phenotypes, excluding the possibility of off-target effects (Fig. S4C). Inversely, the overexpression of RPL35 and DRAP1 in THP-1 macrophages decreased overall infectivity, even for the SERINC5-lacking viruses (Fig. S4D). The loss of RPL35 and DRAP1 in macrophages also rescued the viral transcriptional block (Fig. 5H) in conditions under which the integration of proviral DNA remained unaltered (Fig. S4E). This rescue followed an improvement in the levels of intracellular viral capsid (Fig. 5I).

RPL35 is an mRNA-binding protein (27). We assessed whether this canonical property of RPL35 contributed to the HIV-1 transcriptional defect via differential interaction with viral mRNA when cells were infected with HIV-1 (SERINC5+). However, photoactivatable ribonucleoside enhanced crosslinking and immunoprecipitation (PAR-CLIP) experiments revealed a similar association of HIV-1 mRNA with RPL35 in HIV-1 (SERINC5+/−)-infected cells (Fig. S4F). DRAP1, on the other hand, is a known transcriptional repressor associated with DR1, which interacts with the TBP-TATA complex and prevents the binding TFIIIB required for RNA polymerase (pol) II-mediated transcription (28). Therefore, we assessed whether it was the differential binding of DRAP1 to the long terminal repeat (LTR) core promoter in cells infected with HIV-1 (SERINC5+) to cause the observed transcriptional repression. The chromatin immunoprecipitation (ChIP) assay, however, did not reveal any significant differences in the LTR association with DRAP1 (Fig. S4G). These experiments confirmed the involvement of host proteins RPL35 and DRAP1, albeit noncanonically, in mediating the observed effects.

### MCE1 interacts with RPL35 and DRAP1 in HIV-1 (SERINC5+)-infected macrophages.

**FIG 6:**
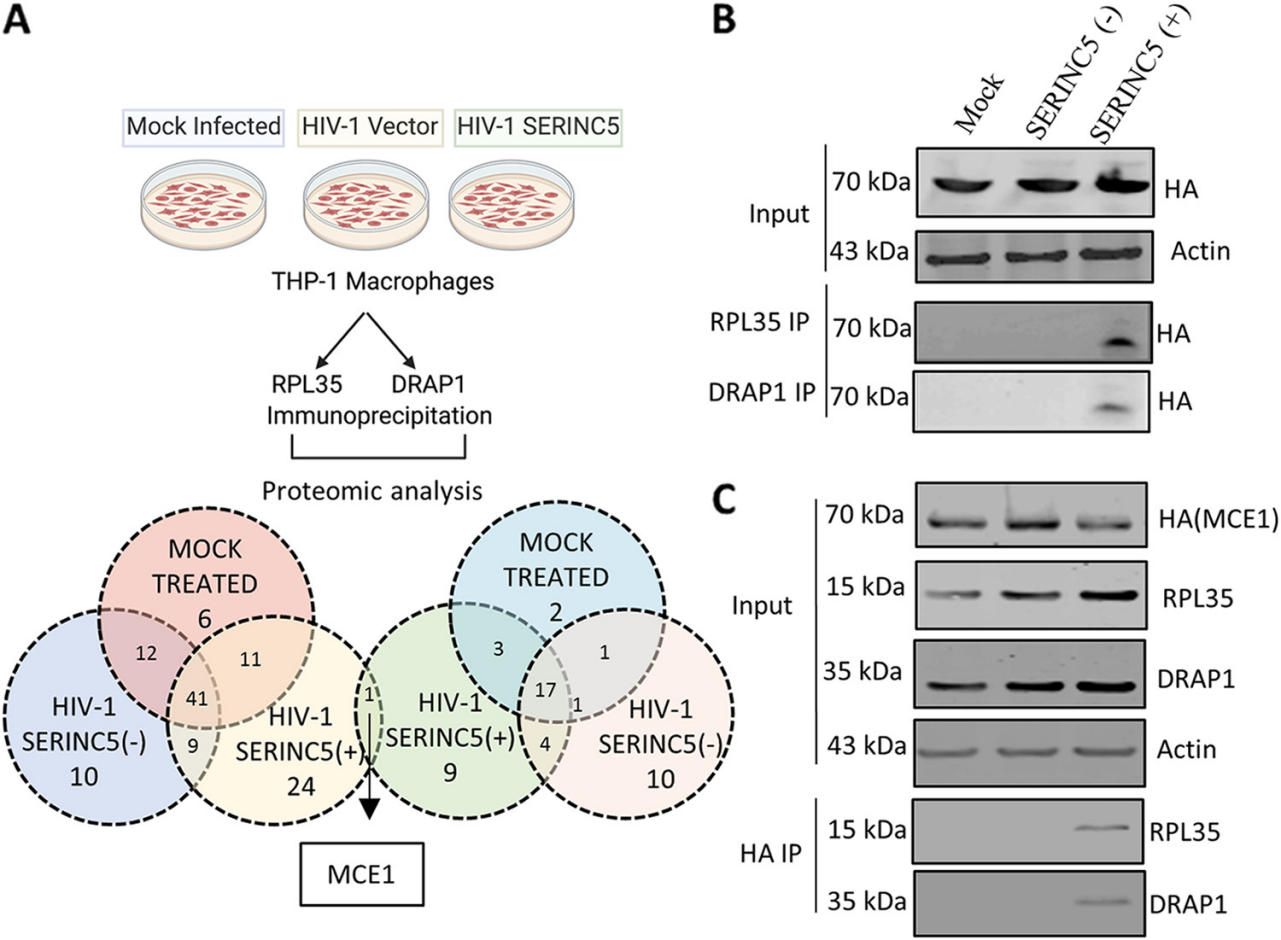
Analysis of RPL35 and DRAP1 protein interactome. (A) Experimental setup and analysis of liquid chromatography-mass spectrometry (LC-MS) data acquired after immunoprecipitation (IP) of RPL35 and DRAP1. (B) Detection of C-terminally HA-tagged MCE1 and corresponding β-actin from the input lysates and from the RPL35 and DRAP1 immunoprecipitates from infected THP-1 macrophages by Western blotting. THP-1 stably expressing MCE-1-HA were generated by lentiviral transduction and were either mock-infected or infected with HIV-1 SERINC5+/− produced from HEK293T. (C) Reciprocal detection by Western blotting of C-terminally HA-tagged MCE1, RPL35, DRAP1, and the corresponding β-actin from input lysate and MCE1 HA immunoprecipitates obtained from infected MCE1-HA-expressing THP-1 macrophages.

Since the canonical roles of RPL35 and DRAP1 at the mRNA and chromatin levels, respectively, did not explain the reduced cytoplasmic abundance of viral RNA and viral protein synthesis, we sought to perform a protein interactome analysis to capture mutual interactions that could help us delineate the mechanism. Intriguingly, our integrative analysis revealed a mammalian capping enzyme (MCE1) as the candidate associated with both RPL35 and DRAP1 only in cells infected with HIV-1 (SERINC5+) (Fig. 6A; Table S3). We therefore generated a THP-1 cell line stably expressing MCE1-hemagglutinin (HA) (Fig. S5A) that allowed us to biochemically confirm this association by detecting HA-tagged MCE1 in immunoprecipitates of RPL35 and DRAP1 (Fig. 6B). Reciprocally, MCE1-HA pulldown indeed detected RPL35 and DRAP1 (Fig. 6C), confirming the specificity of these interactions and the interactome remodeling induced by SERNIC5+ virions.

10.1128/mbio.00166-23.5 FIG S5 SERINC5 abrogates MCE1 and Tat interaction. (A) Western blot showing MCE1 HA expression from a lentivirally transduced stable cell line and corresponding β-actin in THP-1 cells. (B) mRNA expression of MCE1 quantified by qPCR in MCE1 knockdown THP-1 cells generated. (C) RT-normalized infectivity of Nef-defective, SERINC5+/− HIV-1 in THP-1 macrophages indicated knockdown conditions. (D) Western blot showing MCE1 overexpression in THP-1 and corresponding β-actin from the cell lysate. The bands are quantified on Image studio. Lower panel, RT-normalized infectivity of Nef-defective, SERINC5+/− HIV-1 in corresponding MCE1-overexpressed THP-1 macrophages. (E) Schematic of the interaction of MCE1 with Tat and RNA pol II. (F) TAR locus enrichment with MCE1 in the presence and absence of Tat quantified by ChIP assay. (G) Western blot showing RNA polymerase (pol II) and the corresponding β-actin from input lysate and detection of RNA pol II from the pulldown fraction from MCE1 HA immunoprecipitation from infected THP-1 macrophages. (H) RT-normalized infectivity of lentiviral vectors produced in the presence or absence of SERINC5 in THP-1 macrophages. (I) mRNA expression of RPL35 and DRAP1 in THP-1 macrophages infected with lentiviral vector particles produced in the presence or absence of SERINC5, with or without Tat. The values are means (*n* = 3) and SD. An unpaired *t* test was used. *, *P* < 0.05; **, *P* < 0.01; ***, *P* < 0.001; ns, not significant. Download FIG S5, TIF file, 0.9 MB. Copyright © 2023 Ramdas and Chande. 2023 Ramdas and Chande. https://creativecommons.org/licenses/by/4.0/ This content is distributed under the terms of the Creative Commons Attribution 4.0 International license.

Next, we assessed whether genetic depletion of MCE1 would recapitulate the decreased viral transcript abundance observed with HIV-1 (SERINC5+)-infected cells. MCE1 knockdown, however, was accompanied by poor cell survival, and we could not obtain a cell population in which MCE1 was depleted by more than 50% (Fig. S5B) to address this directly. At this level of knockdown, a significant effect on infectivity was also not seen (Fig. S5C). However, upon overexpressing MCE1 at different levels in THP-1, we find that the infectivity defect caused by HIV-1 (SERINC5+) is rescued (Fig. S5D). These experiments revealed specific protein-protein interactions assembled in response to challenge from SERINC5-incorporated virions.

### Loss of MCE1 and Tat interaction reduces HIV-1 mRNA capping.

**FIG 7:**
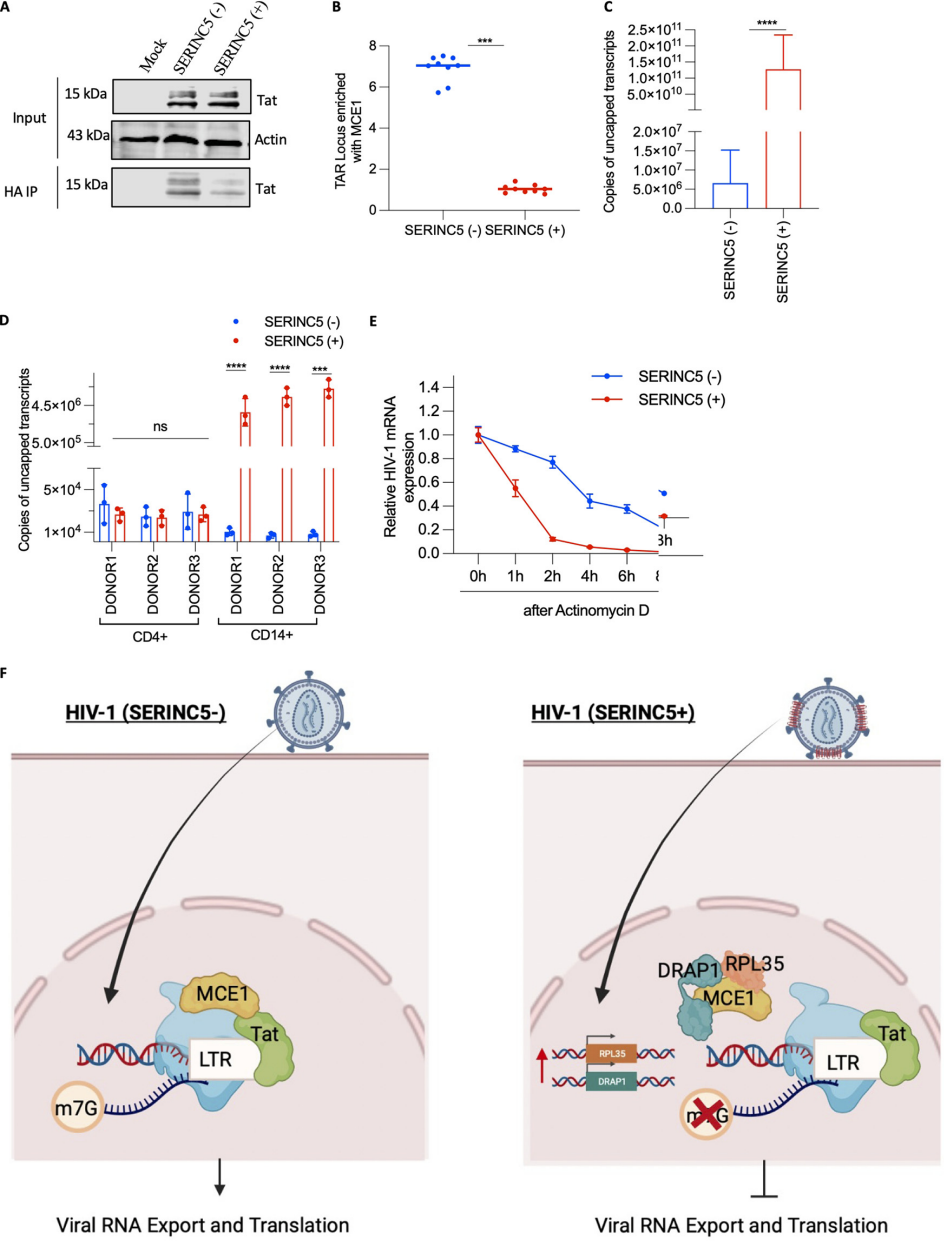
MCE1 and Tat interaction and its effect on viral RNA capping. (A) Detection by Western blotting of HIV-1 Tat and corresponding β-actin from the input lysates and MCE1 HA immunoprecipitates from MCE-1 HA-expressing THP-1 macrophages that were either mock-infected or infected with SERINC5+/− HIV-1 produced by cotransfecting VSV-G, NL4-3, Luciferase Env(−) R(−), SERINC5, or the equivalent empty vector from HEK293T. (B) MCE-1 ChIP assay from MCE1-HA-expressing THP-1 macrophages infected with Nef-defective SERINC5+/− HIV-1 as in panel A depicting Trans-activation response (TAR) region enrichment. The values are means (*n* = 3) and SD. An unpaired *t* test was used. (C) Uncapped transcripts quantified using RNA-ligation-mediated reverse transcription qPCR from THP-1 macrophages infected with Nef-defective HIV-1 produced as in panel A. The values are means (*n* = 3) and SD. An unpaired *t* test was used. (D) Uncapped transcripts in infected CD4^+^ T cells and CD14^+^-derived macrophages of three donors measured as in panel c. The values are means (*n* = 3) and SD. An unpaired *t* test was used. (E) HIV-1 mRNA stability measured by qPCR after actinomycin D treatment at the indicated time points in THP-1 macrophages infected as in panel A. The values are means (*n* = 3) and SD. An unpaired *t* test was used. *, *P* < 0.05; **, *P* < 0.01; ***, *P* < 0.001; ****, *P* < 0.0001; ns, not significant. (F) A plausible model for SERINC5-mediated regulation of viral gene expression in macrophages. LTR, long terminal repeat.

The interaction of HIV-1 Tat and MCE1, *in vitro* as well as in experiments from HeLa cells, has been implicated for efficient cotranscriptional capping of HIV-1 RNA (29, 30) (Fig. S5E). Considering these reports, we investigated the Tat-MCE1 axis in THP-1 macrophages infected with HIV-1 (SERINC5+/−). First, we confirmed with ChIP that in the absence of Tat, MCE1 association with the Trans-activation response (TAR) region of HIV-1 is hampered. For this, cells were transfected with LTR-zsgreen and MCE1-HA-expressing plasmids, and chromatin immunoprecipitation was performed for HA-tagged MCE1 (Fig. S5F). These results reinforced the requirement of Tat for MCE1 recruitment to the HIV-1 transcriptional complex in macrophages. Next, we immunoprecipitated interacting proteins of MCE1 in HIV-1 (SERINC5+/−)-infected THP-1 macrophages and detected Tat by Western blotting. Interestingly, we found relatively lower Tat levels associated with MCE1 in macrophages infected with HIV-1 (SERINC5+) (Fig. 7A). Since MCE1 also partners with RNA polymerase II for the capping of host mRNAs, we checked how SERINC5-induced changes affected their association and found that it was hardly affected (Fig. S5G). However, since MCE1 associates with RNA pol II to cap the cellular transcripts globally, it would prove challenging to examine their interaction in the viral transcriptional complex. Therefore, we performed a ChIP assay with MCE1 and quantified its association with the TAR locus and observed increased TAR enrichment with MCE1 in macrophages infected with HIV-1 (SERINC5−) in comparison to cells infected with HIV-1 (SERINC5+) (Fig. 7B). Altogether, these experiments pointed to a specific mechanism in cells infected with HIV-1 (SERINC5+) that dictated the extent of MCE1 association with the HIV-1 transcriptional complex in conditions under which Tat steady-state levels remained unchanged.

Since the impairment of Tat-MCE1 interaction would only affect Tat-dependent transcription, we also produced SERINC5+/− lentiviral vector particles (LVs) containing a Cyclophilin promoter-driven reporter (Tat-independent). The results revealed that SERINC5 did not affect the transcription of the reporter, and the block pertained only to Tat-dependent transcripts (Fig. S5H). Furthermore, learning that only Tat-dependent transcription was influenced by SERINC5, we next questioned whether Tat was required for SERINC5 to upregulate the host genes, RPL35 and DRAP1. Accordingly, we produced SERINC5+/− vesicles and LVs in the presence and absence of Tat. The mRNA expression of RPL35 and DRAP1 was then checked from THP-1 macrophages subjected to these vesicles and LVs. From our previous experiments, we knew that the viral core was required for SERINC5 to exert the infectivity block (Fig. 2A). In this experiment, we additionally find that Tat is also necessary since only in the presence of SERINC5, core, and Tat do we observe the induction of RPL35 and DRAP1 (Fig. S5I). Together, the results obtained indicate that virion-associated SERINC5 requires the viral core and Tat expression in the virus-producing cells to upregulate RPL35 and DRAP1, which then interact with MCE1, leading to reduced association with Tat.

According to the capping checkpoint model, RNA pol II pauses at promoter-proximal positions and awaits cotranscriptional capping of the mRNA after 17 to 20 nucleotides of transcript synthesis (31). Uncapped or incompletely capped transcripts either undergo degradation or affect splicing and nuclear export (32, 33). The cooperation of MCE1 and Tat is essential for capping HIV-1 mRNA. Since this interaction is abrogated in cells infected with HIV-1 (SERINC5+), we postulated that lower capping efficiency was responsible for lower viral RNA detected in the cytoplasm and reflected in the curtailed translation efficiency. To test this hypothesis, we performed an mRNA capping assay to quantify the capping of HIV-1 RNA by RNA-ligation-mediated reverse transcription-PCR (detailed in the Materials and Methods section). We found that in similar experimental conditions, there was ~4 log more uncapped HIV-1 RNA in cells infected with HIV-1 (SERINC5+) in comparison to cells infected with HIV-1 (SERINC5−) (Fig. 7C).

Furthermore, the reduction in viral RNA capping is readily reversed in the presence of Nef (Fig. S6A). Correspondingly, CD14^+^-derived macrophages also displayed higher copies of uncapped viral transcripts in primary macrophages infected with HIV-1 (SERINC5+) (Fig. 7D). Fewer primary CD14^+^ cells infected (compared to THP-1) could explain the difference in the magnitude of effects observed in these experimental conditions. Similar results were obtained when viruses incorporating endogenous SERINC5 were used for infection (Fig. S6B).

10.1128/mbio.00166-23.6 FIG S6 SERINC5 affects the capping of HIV-1 RNA. (A) Copies of uncapped transcripts of HIV-1 from THP-1 macrophages infected with indicated viruses, quantified using RNA-ligation-mediated reverse transcription qPCR. (B) Copies of uncapped transcripts of HIV-1 from THP-1 macrophages infected with viruses produced from Jurkat TAg and Jurkat TAg^SER5−/−^, quantified using RNA-ligation-mediated reverse transcription qPCR. Lower panel, Western blot of input Tat, MCE1, and β-actin and blot of Tat from immunoprecipitation of MCE1-HA. (C) Copies of uncapped transcripts of HIV-1 from infected wild type and RPL35 and DRAP1-overexpressed THP-1 macrophages, quantified using RNA-ligation-mediated reverse transcription qPCR. Right panel, western blot showing RPL35, DRAP1, and the corresponding β-actin from WT and RPL35 and DRAP1-overexpressed cells in indicated infection conditions. (D) Western blot showing RPL35, DRAP1, Tat, MCE1-HA, and β-actin in the input fraction and RPL35, DRAP1, and Tat in the MCE1-HA immunoprecipitated fraction from THP-1 macrophages. (E) Western blot showing Tat from MCE1-HA immunoprecipitation fraction and Tat, MCE1-HA, and β-actin in the input fraction from infected wild type and RPL35 and DRAP1 knockout THP-1 macrophages. (F) Copies of uncapped transcripts of HIV-1 from infected wild type and RPL35 and DRAP1 knockout THP-1 macrophages, quantified using RNA-ligation-mediated reverse transcription qPCR. (G) Flow cytometry analysis of primary CD3+/CD4+ T cells stained with anti-CD3 allophycocyanin (APC) and anti-CD4 fluorescein isothiocyanate (FITC) antibodies and primary CD14+ cells stained with anti-CD14 APC antibody. The values are means (*n* = 3) and SD. An unpaired *t* test was used. *, *P* < 0.05; **, *P* < 0.01; ***, *P* < 0.001; ns, not significant. Download FIG S6, TIF file, 1.4 MB. Copyright © 2023 Ramdas and Chande. 2023 Ramdas and Chande. https://creativecommons.org/licenses/by/4.0/ This content is distributed under the terms of the Creative Commons Attribution 4.0 International license.

10.1128/mbio.00166-23.7 FIG. S7 Western blots corresponding to the figures indicated. Download FIG S7, PDF file, 1.4 MB. Copyright © 2023 Ramdas and Chande. 2023 Ramdas and Chande. https://creativecommons.org/licenses/by/4.0/ This content is distributed under the terms of the Creative Commons Attribution 4.0 International license.

Since RPL35 and DRAP1 interact with MCE1 exclusively during HIV-1 (SERINC5+) infection, we evaluated whether the expression levels of these host genes affected the viral RNA capping and found that overexpression of the host genes reduced the viral RNA capping even for HIV-1 (SERINC5−) (Fig. S6C). This can be explained by the fact that overexpression of RPL35 and DRAP1 in THP-1 macrophages led to decreased interaction of MCE1 with viral protein Tat and correspondingly increased interaction with the host genes (Fig. S6D). Conversely, in RPL35 and DRAP1 knockout THP-1 cells, the reduced interaction of Tat and MCE1 is restored (Fig. S6E) and thus shows fewer uncapped viral transcripts when infected with HIV-1 (SERINC5+) (Fig. S6F). Furthermore, we checked the HIV-1 mRNA stability in infected cells using actinomycin D to halt transcription and noticed that viral RNA had lower stability when cells were infected with HIV-1 (SERINC5+) (Fig. 7E). Altogether, our experiments suggest that in response to the challenge of SERINC5+ viruses, MCE1 interactome remodeling results in disruption of Tat interaction, which leads to reduced viral RNA capping and gene expression (Fig. 7F). To disrupt MCE1 interaction with Tat, virion-incorporated SERINC5 induces the expression of RPL35 and DRAP1 through yet-unknown intermediates. These host genes then compete with Tat for MCE1 binding.

## DISCUSSION

We demonstrated that the challenge of macrophages with HIV-1 (SERINC5+) results in a defect in viral gene expression. This inhibitory effect is exerted after the integration of the proviral DNA and results in lower viral transcript abundance in the cytoplasm and reduces subsequent virion biogenesis from macrophages. Under these conditions, however, the challenge of T-cell lines, primary T cells, or the TZM-GFP reporter cells do not exhibit such disparity in intracellular viral transcript abundance. The specificity in monocytes and macrophages in inhibiting the transcription posed a striking question, considering they are one of the natural targets of HIV-1. When infected by SERINC5-incorporated viruses, a distinct expression profile is documented in macrophages derived from THP-1 and primary CD14^+^ monocytes, with a moderate induction of host genes RPL35 and DRAP1. RPL35 is a ribosomal protein that functions in translation initiation and is also an mRNA-binding protein (34, 35). In the context of HIV, RPL35 has been shown to interact with Gag (36); however, we find that this interaction is SERINC5 independent. DRAP1, on the other hand, is a known transcriptional corepressor that functions with its partner DR1. It competitively binds to the Tata-binding protein(TBP) and prevents the interaction of transcription factors with the TBP (28). The TBP binds to all TATA boxes and also to the HIV LTR core promoter (37). We deduced that DRAP1 was not accountable for the decreased transcripts quantified since the association of DRAP1 with LTR remains unchanged in cells infected with HIV-1 (SERINC5+) and HIV-1 (SERINC5−). While the chain of events that leads to the upregulation of RPL35 and DRAP1 upon HIV-1 (SERINC5+) challenge remains to be investigated, and our experiments further reveal that these proteins perform noncanonical functions. Remarkably, the effect observed is coupled with the change in protein-protein interactions that prevent the capping enzyme from being associated with the HIV-1 transcriptional complex, thereby reducing the copies of translationally-competent viral mRNAs.

HIV-1 Tat has been shown to promote viral RNA capping by recruiting MCE1 to the HIV-1 transcriptional complex for cotranscriptional capping (29). With the involvement of MCE1, it was crucial to assess the capping status of viral RNAs. Capped transcripts enhance mRNA stability by preventing the access of decapping enzymes and RNA decay machinery. In contrast, uncapped nascent transcripts recruit RNA surveillance and degradation machinery for their default fate (38). Nuclear-retained RNAs are degraded by these RNA decay enzymes, which is consistent with our experiments.

Based on the evidence from our experiments, we find that along with SERINC5, the viral core is essential for the upregulation of RPL35 and DRAP1. Only upon upregulation of these genes are they able to compete with Tat for interacting with MCE1. Admittedly, we are yet to understand the upstream mechanism by which SERINC5 upregulates these host genes. We speculate that SERINC5, in association with the virus, relays signaling from either the plasma membrane or the cytoplasm that then affect nuclear events such as gene regulation, leading to interactome remodeling at the HIV-1 transcription complex. However, for now, we know that SERINC5 and the virion core are essential for this signaling to be active. We have also shown that Tat binding to MCE1 is lost only in the case when the infection is initiated with SERINC5 incorporated virus and upon upregulation of RPL35 and DRAP1. The possibilities for the loss of this interaction include changes in protein abundance that could have titrated away Tat. Alternatively, it could be a shared binding site on MCE1 that limited the number of possible simultaneous interactions. Finally, if there were simultaneous interactions, RPL35 and DRAP association might have made the Tat binding inaccessible due to the sheer size or a post-translational modification that alters the affinity. While the exact mechanism of this interactome remodeling awaits further characterization, our experiments imply that SERINC5 can exhibit an antiviral effect by controlling the capping and stability of HIV-1 RNAs in macrophages. Co-option of MCE1 by viruses (39) for capping the viral RNA thus represents a vulnerability, as the unavailability of MCE1 would lead to nonproductive infection.

HIV-1 envelope glycoproteins are under strong selection pressure due to the host immune system. It appears that the majority of envelopes from transmitted-founder viruses can resist SERINC5 action (15). SERINC5 mediating a postentry block to viral gene expression is a plausible mechanism that the host can employ to counter retroviral envelope-mediated resistance arising from sequence divergence. Nef, therefore, might ensure the exclusion of SERINC5 from virion incorporation to relieve such transmission bottlenecks in myeloid lineage cells. It will be intriguing to explore whether virion-incorporated SERINC5 is the danger signal recognized by a yet-unknown upstream receptor on macrophages that triggers signaling to specifically target an event as crucial as viral RNA capping. Given that viruses of unrelated families encode factors to prevent SERINC5 virion incorporation, investigating the existence of such upstream receptors/mechanisms in macrophages influencing MCE1 function would be relevant and can form the basis for future studies.

## MATERIALS AND METHODS

### Plasmids and reagents.

Table S1 mentions all the plasmids and reagents used in this study.

### Cell lines and culture conditions.

HT1080 (American Type Culture Collection [ATCC]) (fibroblast), HEK293T (ECACC) (epithelial-like), A549 (ATCC), and TZM-GFP (4) (epithelial) were grown in Dulbecco’s modified Eagle medium containing 10% heat-inactivated fetal bovine serum (FBS) (US Origin) with 2 mM l-GlutaMAX. THP-1 (ATCC), THP-1^SER5−/−^, THP-1 Dual (Invivogen) (monocyte), Jurkat E6.1 (ATCC), Jurkat TAg (4), Jurkat TAg^SER5−/−^ (4), Jurkat^SER3/5−/−^ (17), K562 (National Centre for Cell Science [NCCS]) (lymphoblast) were grown in RPMI 1640 containing 10% heat-inactivated FBS (US Origin) with 2 mM l-GlutaMAX and 10 mM HEPES. THP-1 cells were differentiated into macrophages with 30 ng/mL PMA for 24 h. The cells were maintained in a humidified incubator with 5% CO_2_ at 37°C. All the cell lines were tested for mycoplasma and were found to be negative.

### Viruses and infectivity.

Single-cycle viruses were produced from HEK293T cells by transfecting 1 μg envelope glycoprotein (vesiculostomatitis virus glycoprotein [VSV-G] encoding pMD2.g [Table S1A] [3], JR-FL env [Table S1A] [31] or HXB2 [Table S1A] [4]), 7 μg NL4-3, Luciferase Env(−), R(−) (Table S1A) (1), or NLBNzsgreen (a derivative of NL4-3 lacking envelope and encodes a zsgreen fluorescent protein from the Nef locus) (Table S1A) (2) and 1 μg plasmid encoding SERINC5 (Table S1A) (6) or the equivalent empty vector in a 10 cm plate. For SERINC5 counteraction studies, 1 μg of PBJ6 SERINC5 (Table S1A) (5) and 1 μg of either Nef clade C (Table S1A) (8) or Nef Lai (Table S1A) (11) and indicated Nef mutants (Table S1A) (12–15) encoded by PBJ5 or pcDNA encoded MLV GlycoGag (Table S1A) (9) were cotransfected during virus production. The supernatant containing viral particles was collected 48 h post-transfection after centrifugation and filtered through a 0.22-μm syringe filter to remove cell debris. The viruses were quantified by the SGPERT assay (in brief, below). Equal volumes of viruses produced under different conditions were added to the target cells. The infected cells were either lysed and processed for luciferase quantification as described earlier (40) or were analyzed by scoring zsgreen-positive cells on a cell-imaging multimode reader (Molecular Devices), depending on the reporter virus used. The obtained reporter readouts were normalized to the reverse transcriptase (RT) units from the SGPERT assay, and the infectivity from HIV-1 (SERINC5−) was set to 100% for comparison with HIV-1 SERINC5(+). The following formula was used for converting raw infectivity values to percentage infectivity:

$$\text{Infectivity}\;\text{units}\;\left(\frac{\text{I.U.}}{\text{mu}\;\text{RT}}\right)=\frac{\text{Avg}\;\text{no.}\;\text{of}\;\text{infected}\;\text{cells}\times 1,000,000}{\text{RT}\;\text{value}\times \text{volume}\;\text{of}\;\text{virus}\;\text{added}}$$

$$\text{Infectivity}\;\left(\%\right)=\frac{\text{Infectivity}\;\text{units}\;\text{of}\;\text{sample}\times 100}{\text{Infectivity}\;\text{units}\;\text{of}\;\text{vector}}$$

### Sybr green I-based product-enhanced reverse transcriptase (SGPERT) assay.

After collecting and filtering the virus-containing supernatant, 5 μL of the virus was mixed with 5 μL of lysis buffer (0.25% Triton X-100, 50 mM KCl, 100 mM Tris-HCl, pH 7.4, 40% glycerol). After incubation for 10 min at room temperature, the reaction was diluted with 90 μL 1× core buffer (5 mM [NH_4_]_2_SO_4_, 20 mM KCl, and 20 mM Tris-HCl, pH 8.3). From this mixture, 10 μL was added to 10 μL 2× reaction buffer (5 mM [NH_4_]_2_SO_4_, 20 mM KCl and 20 mM Tris-Cl, pH 8.3, 10 mM MgCl_2_, 0.2 mg/mL bovine serum albumin [BSA], 1/10,000 SYBR green I, 400 μM dNTPs, 1 μM forward primer, 1 μM reverse primer, 7 pmol/mL MS2 RNA). This was subjected to qPCR with the following primers: 5′-TCCTGCTCAACTTCCTGTCGAG-3′ and 5′-CACAGGTCAAACCTCCTAGGAATG-3′. Using a standard with known RT units, the values for the virus were extrapolated (41).

### Incorporation of SERINC5 in virions.

The viruses were produced from HEK293T in a 10 cm plate, as mentioned above. At 48 h post-transfection, the virus-containing supernatant was centrifuged at 300 × *g* for 5 min and filtered using a 0.22-μm syringe filter to remove cell debris. The filtered suspension was overlaid on a 20% sucrose cushion and centrifuged at 100,000 × *g* for 2 h at 4°C on a Beckman-Coulter ultracentrifuge. The pellet was dissolved in 4× Laemmli buffer containing 50 mM Tris(2-carboxyethyl)phosphine hydrochloride (TCEP), and SERINC5 and P24 were detected per the Western blotting protocol (below).

### VSV-G enveloped vesicles production.

HEK293T cells were transfected with either only empty vector (for conditioned media), VSV-G (Table S1A) (4), and empty vector (for Vector vesicles) and VSV-G and SERINC5 (Table S1A) (6) (for SERINC5[+] vesicles). At 48 h post-transfection, the supernatant was collected and filtered. The concentration of the extracellular vesicles was carried out by ultracentrifugation. THP-1 macrophages were primed with the vesicles for 6 h prior to infection with HIV-1 (SERINC5−) and HIV-1 (SERINC5+).

### Lentiviral particle (LV) production.

HEK293T cells in a 10 cm plate were transfected with 2 μg VSV-G (Table S1A) (4), 6 μg psPAX2 (Table S1A) (25), 8 μg pSCalps zsgreen (Table S1A) (39), and 500 ng either empty vector or SERINC5 (Table S1A) (6). The lentiviral particles containing supernatant were collected and filtered using a 0.22-μm syringe filter and added to THP-1 macrophages. The transduced cells were analyzed by scoring the zsgreen-positive cells. The reporter readouts were normalized to RT units obtained from the SGPERT assay.

### Fusion assay.

Fusion was quantified by a nlsCre assay as previously described (4). In brief, nlsCre viral particles were produced by transfecting HEK293T cells with 8.9 Cre (Table S1A) (27) along with VSV-G (Table S1A) (4) and plasmids encoding SERINC5 (Table S1A) (6) or equivalent empty vector. THP-1 cells stably expressing an RFP reporter (Cre-induced) were challenged with the produced viruses, and 48 h later, RFP-positive cells were quantified. The cells were treated with either dimethyl sulfoxide (DMSO), bafilomycin (100 nM), or AZT (10 μM) 1 h prior to infection.

### Subcellular fractionation.

For subcellular fractionation, the cells were processed after infection as previously described (42). Briefly, the cells were lysed in a hypotonic buffer containing 10 mM Tris-HCl (pH 7.5), 10 mM NaCl, 3 mM MgCl_2_, 0.5% NP-40, 1× protease inhibitor. After centrifugation at 500 × *g* for 10 min at 4°C, the supernatant was collected to represent the cytoplasmic fraction. The nuclear pellet was washed thrice with hypotonic buffer to reduce endoplasmic reticulum contamination, after which they were lysed with nuclear lysis buffer (50 mM Tris-HCl [pH 8.1], 10 mM EDTA, 1% SDS, 1× protease inhibitor) and then sonicated for 2 cycles for 20 s each at 20% amplitude. The sample was then centrifuged at 14,000 × *g* for 10 min at 4°C.

### Quantification of reverse transcription, integration, and transcription.

Cells infected with the indicated viruses were collected at different time points and lysed for subcellular fractionation. The DNA isolated from the cytosolic fraction was analyzed for reverse transcription products by qPCR (43) using the following primers: Fwd 5′-TCTGGCTAACTAGGGAACCCA-3′ and Rev 5′- CTGACTAAAAGGGTCTGAGG-3′. The late reverse transcription products were quantified at 24 h postinfection from total cell DNA by qPCR using the following primers: Fwd 5′-GACCCTTTTAGTCAGTGTGGAAA-3′ and Rev 5′-CTTCAGCAAGCCGAGTCCT-3′. For integration, DNA isolated from the nuclear fraction was subjected to Alu-Gag PCR to quantify integrated proviral copies (44). In brief, it is a two-step PCR in which the first step, genomic DNA with Alu Fwd 5′-GCCTCCCAAAGTGCTGGGATTACAG-3′ and Gag Rev 5′-TACCATTTGCCCCTGGAGGTT-3′ were used for preamplification. The amplicon from this PCR was used as a template for a second round of qPCR using Fwd 5′-TCTGGCTAACTAGGGAACCCA-3′ and Rev 5′-CTGACTAAAAGGGTCTGAGG-3′ to quantify HIV-1 integration. HIV-1 transcription was measured by the abundance of HIV-1 transcripts present in the cytosolic fraction at different time points. RNA was isolated from the cytosolic fraction using TRIzol, and single-strand cDNA synthesis was performed using the random hexamer primer. The transcripts were quantified by qPCR with the primers Fwd 5′-TTGTACTGAGAGACAGGCT-3′ and Rev 5′-ACCTGAAGCTCTCTTCTGG-3′.

### RNA fluorescence *in situ* hybridization (FISH).

Twenty-nucleotide-long oligonucleotides (*n* = 13) complementary to HIV-1 mRNA were labeled with Alexa 488 using the Ulysis Alexa Fluor 488 nucleic acid labeling kit (Thermo Scientific), as per the manufacturer’s protocol. These oligonucleotides were used as hybridization probes for detecting HIV-1 RNA within infected cells. THP-1 monocytes were grown on coverslips and differentiated into macrophages. These cells were then infected with HIV-1, and 20 h postinfection, the cells were fixed in 4% paraformaldehyde. Following this, the cells were permeabilized with 0.3% Triton X-100. The coverslips were then dipped in a hybridization buffer (50% formamide, 2× SSC buffer [1× SSC is 0.15 M NaCl plus 0.015 M sodium citrate], and 10% [wt/vol] dextran sulfate) containing the probes and incubated overnight at 37°C in a dark and humid chamber. The next day, the cells were thoroughly washed with 1× phosphate-buffered saline (PBS) and 4 × SSC buffer before nuclear staining with Hoechst. The coverslips were then mounted on slides and allowed to dry overnight. The cells were imaged on a Zeiss Apotome.2 fluorescence microscope. The image processing and puncta scoring was done on Fiji-2.

### Polysome fractionation.

Twenty-four hours after infection, the cells were treated with cycloheximide (CHX) (100 μg/mL) and incubated for 10 min at 37°C. Following this, the medium was discarded, and the cells were washed thrice with 1× PBS. The cells were then scraped off the surface and lysed in polysome extraction buffer (20 mM Tris-HCl, pH 7.5, 100 mM KCl, 5 mM MgCl_2_, 0.5% NP-40, 1× protease inhibitor, RNase inhibitor, 100 μg/mL CHX). The cytoplasmic fraction was taken for polysome fractionation with 10% to 50% sucrose gradients as previously described (45). Following this, RNA was isolated from the polysome fractions, and cDNA was synthesized as described above. The percentage of remaining mRNA was calculated based on the following equation.

ΔCq=Cq (fraction 1)−Cq (fraction X)

$$\%\;\text{mRNA}\;\text{remaining}=\frac{2^{\Delta \mathrm{Cq}}\times 100}{\sum 2^{\Delta \mathrm{Cq}}}$$

### RNA sequencing and analysis.

A total of 4 μg of total RNA was extracted from mock-treated and HIV-1-infected (SERINC5+/−) THP-1-derived macrophages. RNA-sequencing (RNA-seq) libraries were prepared using the Illumina Truseq Stranded mRNA library prep. Subsequently, the libraries were sequenced on Illumina NovaSeq 6000 with pair-end sequencing. The reads obtained were mapped to the human genome (hg38) using HISAT2 (version 2.1.0) and samtools (version 1.9). Gene expression quantification (read counts) was acquired using HTSeq (version 0.11.3), and differential gene expression profiles were analyzed using DESeq2 on R (version 3.6.3).

### Generation of stable cell lines.

For the generation of stable knockouts of RPL35 and DRAP, lentiviruses were produced by transfecting three guide RNAs and two guide RNAs for SERINC5 against the target gene, respectively, encoded by a lentiviral transfer vector. For stable knockdown of candidate genes, short hairpin RNAs (shRNAs) against the target gene (five each) (Dharmacon) were packaged into lentiviruses. The guide RNA and shRNA sequences can be found in Table S2. To generate MCE1-HA-expressing THP-1 cells, MCE1 was amplified from THP-1 cDNA and cloned in-frame with a C-terminal HA tag in a lentiviral vector (Table S1A) (24). THP-1 cells expressing RFP reporter induced by Cre were generated by producing lentiviruses packaging p-lenti LoxP-Blasti-mRFP (Table S1A) (4, 28). The mentioned lentiviral transfer vectors were packaged with pMD2.g (Table S1A) (4) and psPAX2 (Table S1A) (25). THP-1 monocytes were transduced by spinoculation. At 48 h post-transduction, the cells were subjected to 1 μg/mL puromycin or 5 μg/mL blasticidin, depending on the selection marker, for selecting the transduced population.

### Electroporation.

A total of 10^7^ THP-1 monocytes, Jurkat TAg and Jurkat TAg ^SER5−^ were taken from the log phase of growth for transfection by electroporation. For virus production from Jurkat cells, 1 μg VSV-G (Table S1A) (4) and 7 μg NL4 E-R- Luc (Table S1A) (1) were mixed with the cells resuspended in Opti-MEM. The BTX Gemini SE system was used to provide a single pulse of 230V in LV mode for 65 ms. For transfection in THP-1 cells, 5 μg of RPL35, DRAP1, and MCE1 (Table S1A) (33–35) was added to the cells resuspended in Opti-MEM. A single pulse of 350V in LV mode for 5 ms was provided for electroporation.

### RNA isolation and qPCR.

RNA was extracted using TRIzol as per the manufacturer’s protocol. DNase I treatment was provided to the RNA samples to remove residual DNA contamination. First-strand cDNA synthesis was performed using either oligo(dT) primer or random hexamer using RevertAid reverse transcriptase. SYBR green-based quantitative PCR was performed on the Bio-Rad CFX96 real-time PCR detection system.

### Western blotting.

Cell pellets were lysed in DDM lysis buffer (100 mM NaCl, 10 mM HEPES [pH 7.5], 50 mM TCEP, 1% *n*-dodecyl-β-d-maltoside [DDM], 2× Protease inhibitor cocktail) for SERINC5 detection and in radioimmunoprecipitation assay (RIPA) buffer (supplemented with 2× protease inhibitor cocktail and 50 mM TCEP) for detecting the other proteins. After incubation on ice for 30 min, the lysate was centrifuged at 14,000 × *g* for 15 min at 4°C, and the supernatant was taken and added to an equal volume of 4× Laemmli buffer. The samples were resolved on SDS-Tricine-PAGE. The proteins from the gel were transferred to a polyvinylidene difluoride (PVDF) membrane by electroblotting. The primary and secondary antibodies used are enlisted in Table S1B. The blots were acquired on the Odyssey imager system (LI-COR Biosciences).

### Immunoprecipitation and mass spectrometry.

At 24 h postinfection, the cell culture supernatant was discarded, and disuccinimidyl suberate (DSS) (10 μg/mL) was added to the cells for protein cross-linking. After a 30-min incubation, the cells were thoroughly washed with 1× PBS and lysed in nondenaturing RIPA buffer to keep the interactions intact. The lysate was then incubated with antibody-conjugated protein G beads for 3 h. The beads were then washed, and the proteins were eluted in 4× Laemmli buffer. The samples were then briefly run on an SDS-PAGE gel, and in-gel trypsinization was performed. The peptides were separated on mass spectrometry instrument (ESI-QUAD-TOF), and the peaks were analyzed on the Mascot Server.

### Chromatin immunoprecipitation (ChIP).

A ChIP assay inspected the association of DRAP1 and MCE1 to HIV-1 LTR, as described previously. Briefly, infected THP-1 macrophages were lysed to extract the nuclear fraction and then sonicated using a probe sonicator to acquire fragmentation in the range of 200 to 500 bp. Following this, chromatin immunoprecipitation was done by incubating 25 μg chromatin with DRAP1, HA, or control IgG antibody overnight at 4°C. The chromatin-protein complex was pulled down with protein G Dynabeads. The eluted samples were subjected to RNase treatment and proteinase K digestion. The DNA isolated was analyzed by qPCR. The acquired *Cq* values were normalized to the IgG control.

### Quantification of uncapped mRNA.

A modified protocol of RNA-ligation-mediated reverse transcription followed by DNA amplification was employed for the assay (46). In brief, an RNA anchor was selectively ligated to 5′-monophosphate of mRNA by T4 RNA ligase using a splint DNA (one half complementary to the RNA anchor and the other half complementary to the transcript of interest) commercially synthesized. Capped transcripts would have a 5′-triphosphate and would not be able to ligate with the RNA anchor. cDNA synthesis was performed, and uncapped mRNA was quantified by qPCR with primers flanking the anchor and the gene of interest. The copy number of uncapped transcripts was calculated by extrapolating from a standard curve derived from RNA ligation of *in vitro* transcribed luciferase RNA (uncapped) with the RNA anchor. As a control for capped mRNA, β-actin was taken.

### mRNA stability assay.

THP-1 monocytes were differentiated in three 6-well plates with 30 ng/mL PMA for 24 h. After differentiation, each plate was either infected with mock, HIV-1 produced without SERINC5, and HIV-1 was produced with SERINC5. After 24 h of infection, the cells from one well of each plate were taken for RNA isolation. A total of 10 μg/mL of actinomycin D was added to the rest of the five wells. The cells were collected at 1, 2, 4, and 8 h for RNA extraction, and HIV-1 RNA was quantified by SYBR green-based qPCR (47).

### Primary cells.

Peripheral blood was collected from healthy donors and processed for peripheral blood mononuclear cell (PBMC) isolation. CD3^+^/CD4^+^ cells were obtained from these PBMCs by magnetic beads-based separation using a CD4^+^ isolation kit. The isolated cells were characterized by staining with anti-CD3 allophycocyanin (APC) and anti-CD4 fluorescein isothiocyanate (FITC) antibodies for flow cytometry. These cells were then activated using 5 μg/mL phytohemagglutinin (PHA) and 50 IU/mL recombinant human IL-2 (48). Similarly, CD14^+^ cells were enriched from PBMCs using a CD14^+^ isolation kit. These cells were stained with anti-CD14-APC antibody for flow cytometry (Fig. S6G). CD14^+^ monocytes were differentiated using recombinant human macrophage colony-stimulating factor (M-CSF) for 7 days (49). Both the cells were grown in RPMI 1640 containing 10% human antibody serum, 1× penicillin-streptomycin, 2 mM GlutaMAX, and 10 mM HEPES.

### IRF-3 and NF-κB reporter assays.

THP-1 Dual cells stably express a secretory luciferase reporter for type I interferon (IFN-I) and SEAP reporter for NF-κB. For these assays, the supernatant was collected from infected THP-1 Dual derived macrophages and manufacturer-provided substrates (QuantiLuc and QuantiBlue) were added in injection mode for measuring the luciferase units and colorimetric absorbance at 620 nm on a Spectramax i3x multimode plate reader.

### PAR-CLIP.

THP-1 macrophages (either mock-treated or HIV-1 infected) were subjected to a PAR-CLIP experiment. Concisely, 16 h before cross-linking, 4SU (500 μM) was added to the culture plates. The UV cross-linking was done with 0.2 J/cm^2^ at 365 nm. The cross-linked sample was then processed for pulldown with protein G Dynabeads conjugated with RPL35 antibody by overnight incubation at 4°C. The next day, the beads were washed with high-salt and low-salt buffers before proteinase K digestion. The RNA was then isolated with TRIzol and converted to cDNA for qPCR to measure the protein-associated RNA (50).

### Statistical analysis.

The significance of the results was statistically analyzed using Student’s unpaired *t* test in GraphPad Prism 9. The relative differences between replicates were considered statistically significant in the case of *P* < 0.05. The significance in the results is denoted as **** for *P* < 0.0001, *** for *P* < 0.001, ** for *P* < 0.01, and * for *P* < 0.05 and ns for not significant.

### Data and materials availability.

All of the data are included in this article. The data for the RNA sequencing performed in the study have been deposited with the European Nucleotide Archive under accession number PRJEB53663.

10.1128/mbio.00166-23.9 TABLE S1 List of plasmids and reagents used in the study. Download Table S1, PDF file, 0.04 MB. Copyright © 2023 Ramdas and Chande. 2023 Ramdas and Chande. https://creativecommons.org/licenses/by/4.0/ This content is distributed under the terms of the Creative Commons Attribution 4.0 International license.

10.1128/mbio.00166-23.10 TABLE S2 Sequences of shRNAs and gRNAs used. Download Table S2, PDF file, 0.1 MB. Copyright © 2023 Ramdas and Chande. 2023 Ramdas and Chande. https://creativecommons.org/licenses/by/4.0/ This content is distributed under the terms of the Creative Commons Attribution 4.0 International license.

10.1128/mbio.00166-23.11 TABLE S3 Candidates after mass spectrometry analysis on Mascot Server. Download Table S3, PDF file, 0.16 MB. Copyright © 2023 Ramdas and Chande. 2023 Ramdas and Chande. https://creativecommons.org/licenses/by/4.0/ This content is distributed under the terms of the Creative Commons Attribution 4.0 International license.
