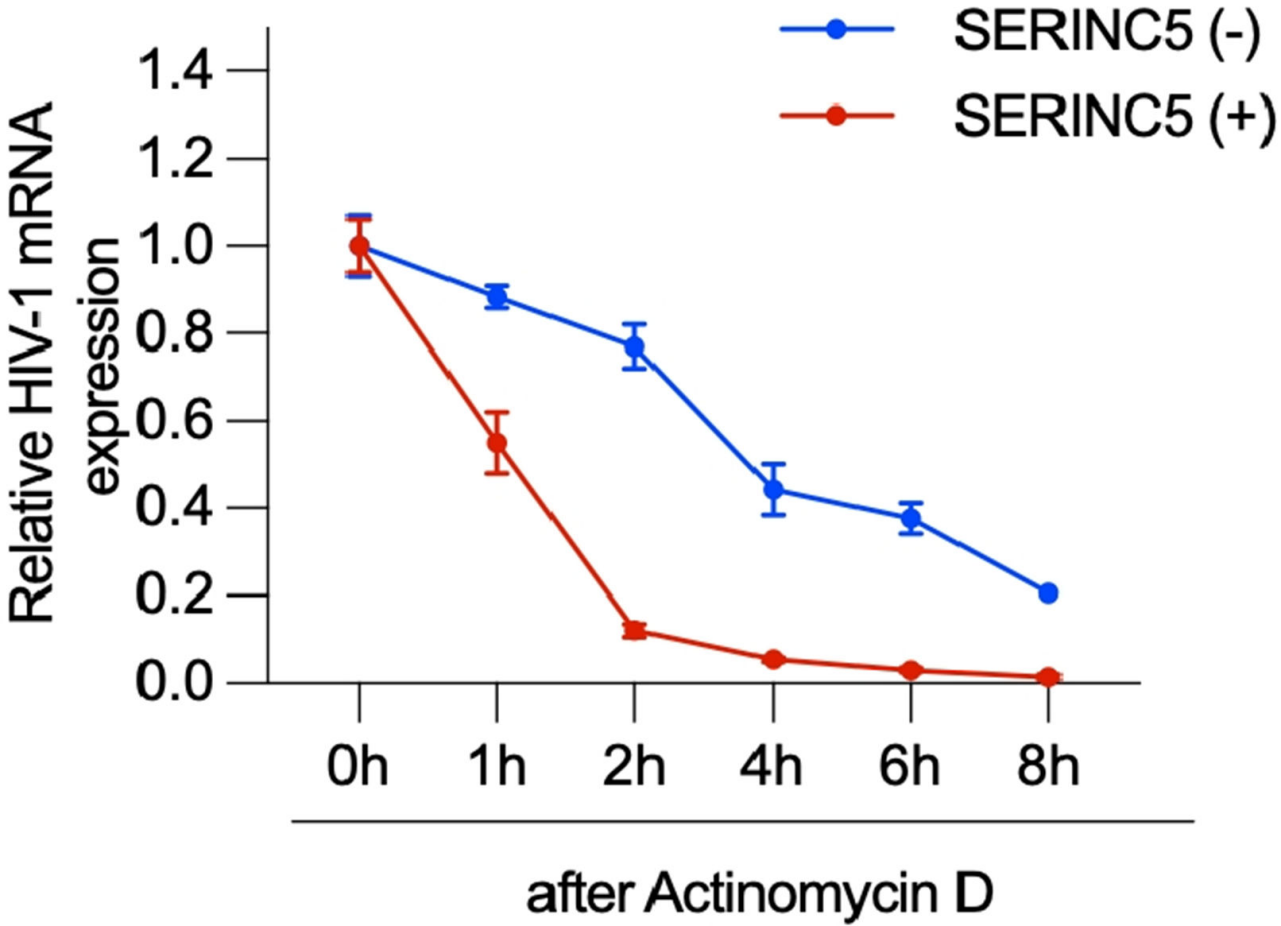# Erratum for Ramdas and Chande, “SERINC5 Mediates a Postintegration Block to HIV-1 Gene Expression in Macrophages”

**DOI:** 10.1128/mbio.01222-23

**Published:** 2023-07-05

**Authors:** Pavitra Ramdas, Ajit Chande

Volume 14, No. 2, e00166-23, 2023, http://doi.org/10.1128/mbio.00166-23. Page 12, Fig. 7: Panel E should appear as shown below.