# Supplementary figures and images for: SERINC5 Mediates a Postintegration Block to HIV-1 Gene Expression in Macrophages
Source: mBio. 2023 Mar 28;14(2):e00166-23. doi: 10.1128/mbio.00166-23 (PMC10127607; doi:10.1128/mbio.00166-23)

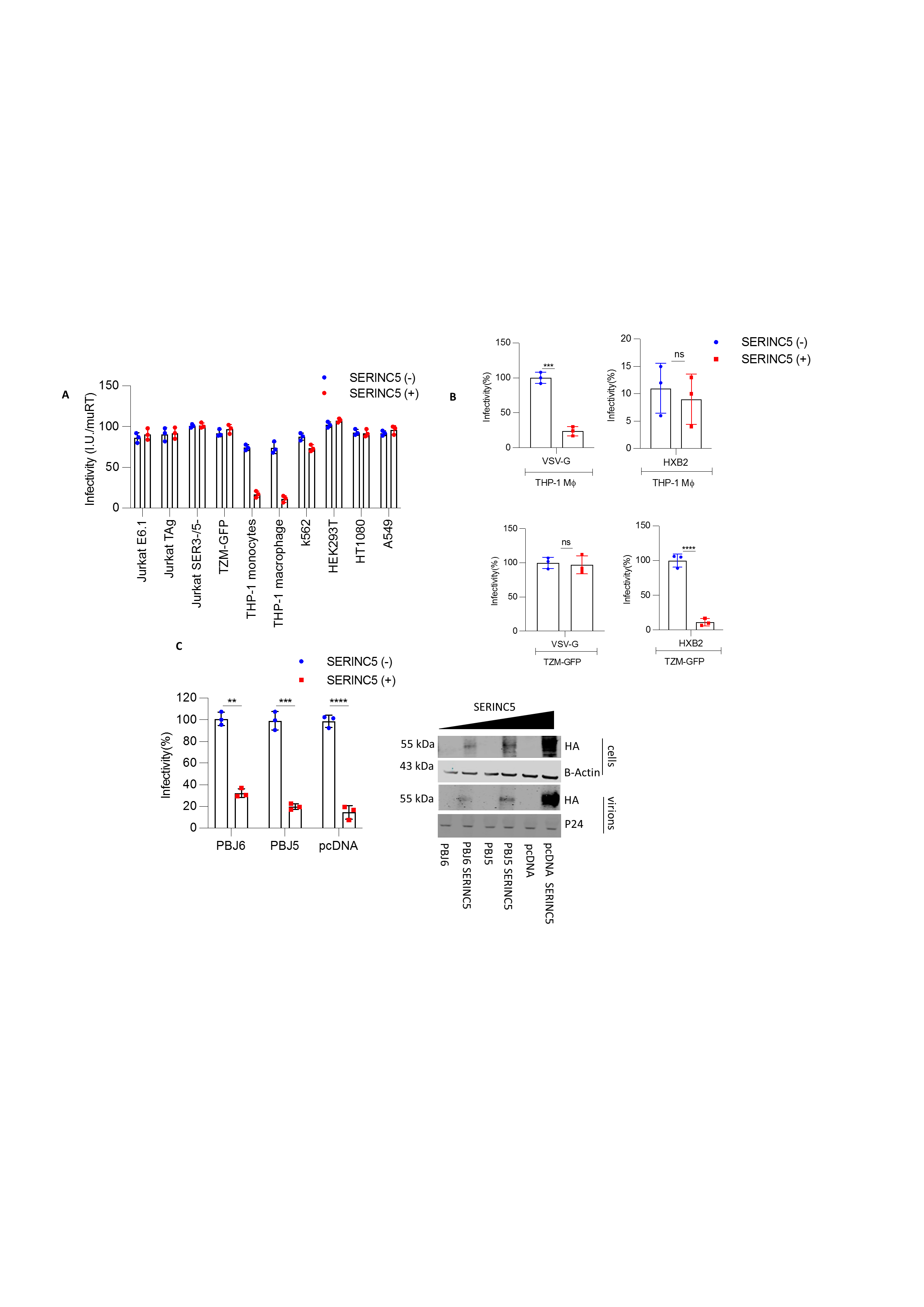

Supplement: FIG S1 [file mbio.00166-23-s0001.tif]

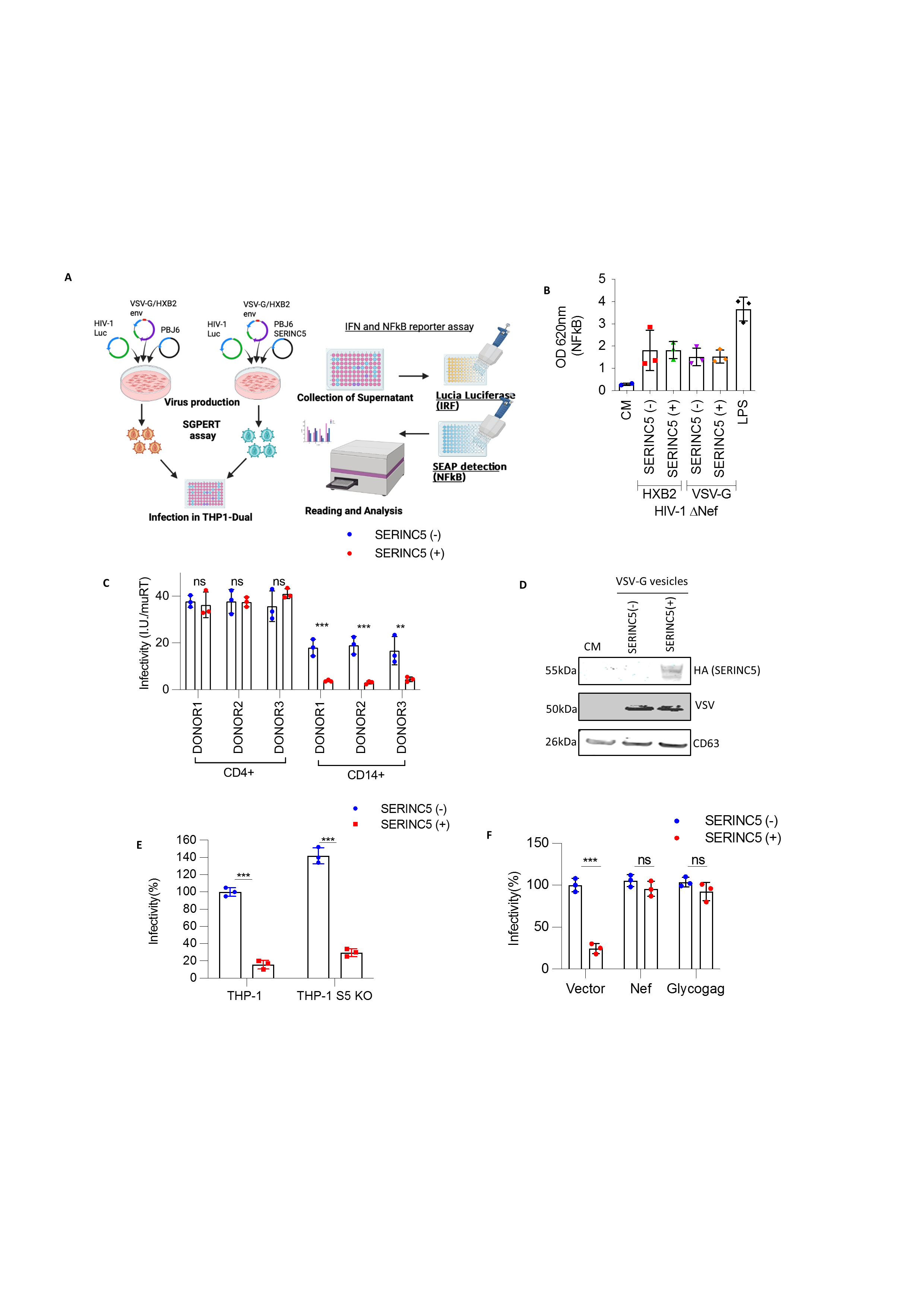

Supplement: FIG S2 [file mbio.00166-23-s0002.tif]

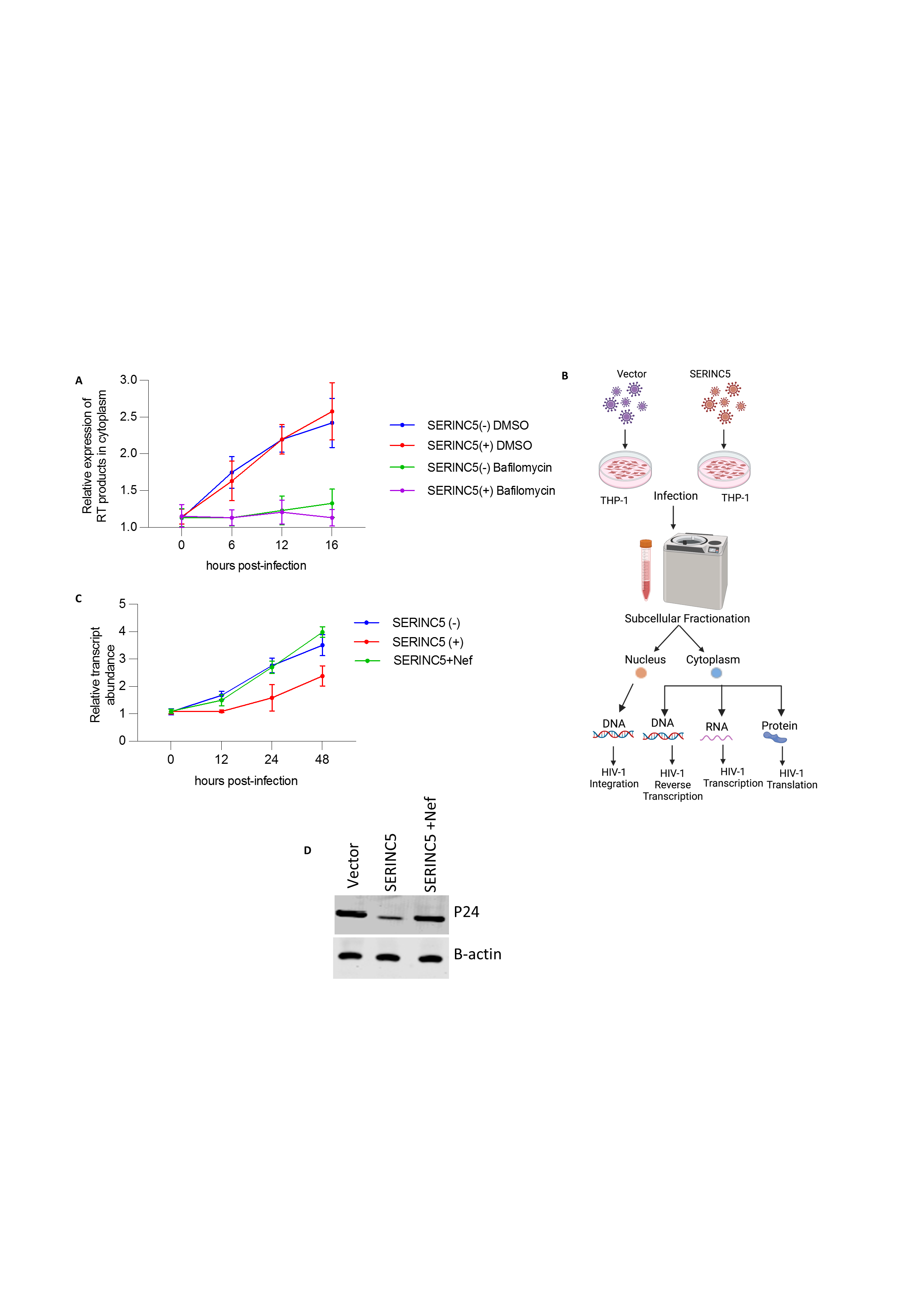

Supplement: FIG S3 [file mbio.00166-23-s0003.tif]

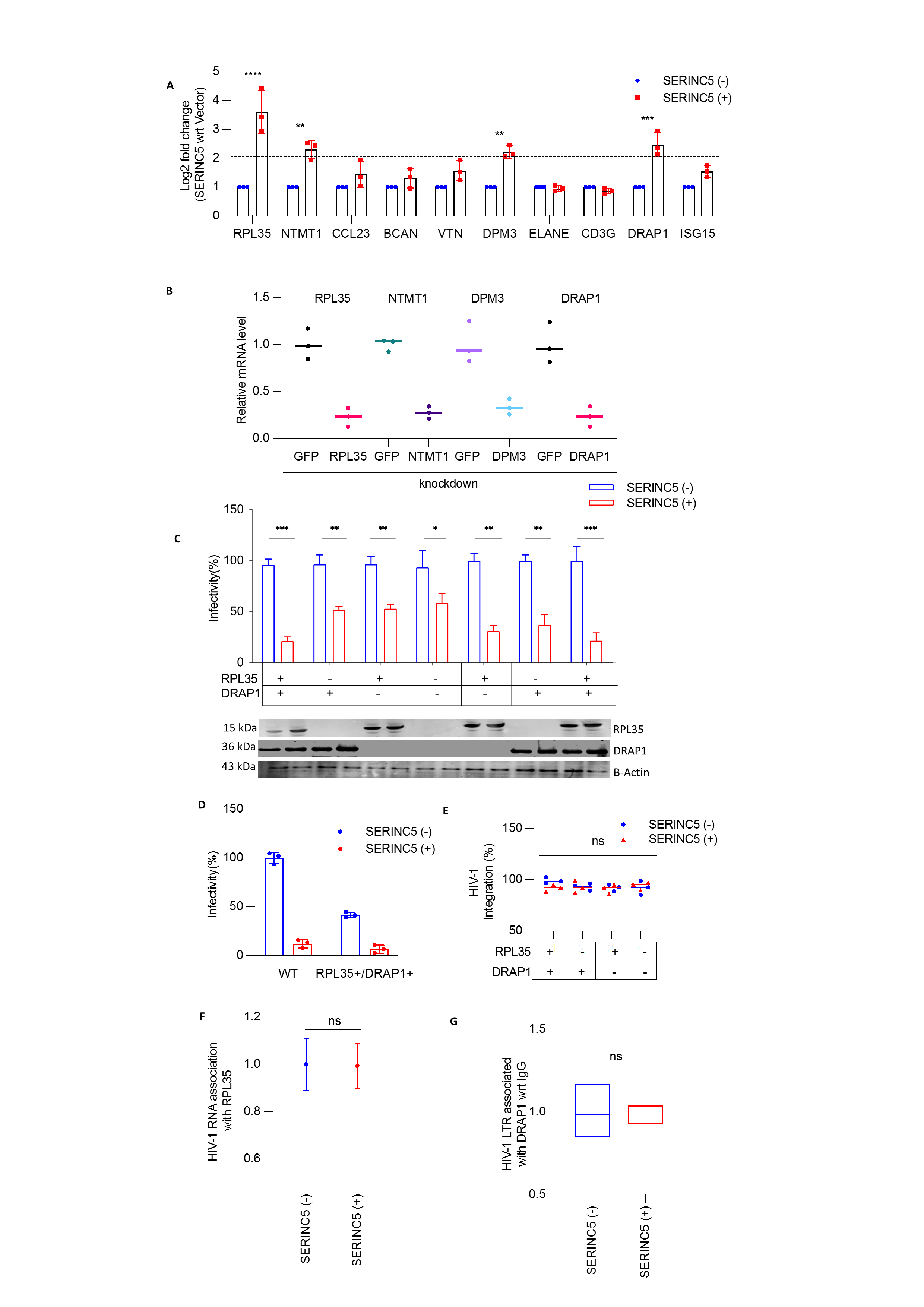

Supplement: FIG S4 [file mbio.00166-23-s0004.tif]

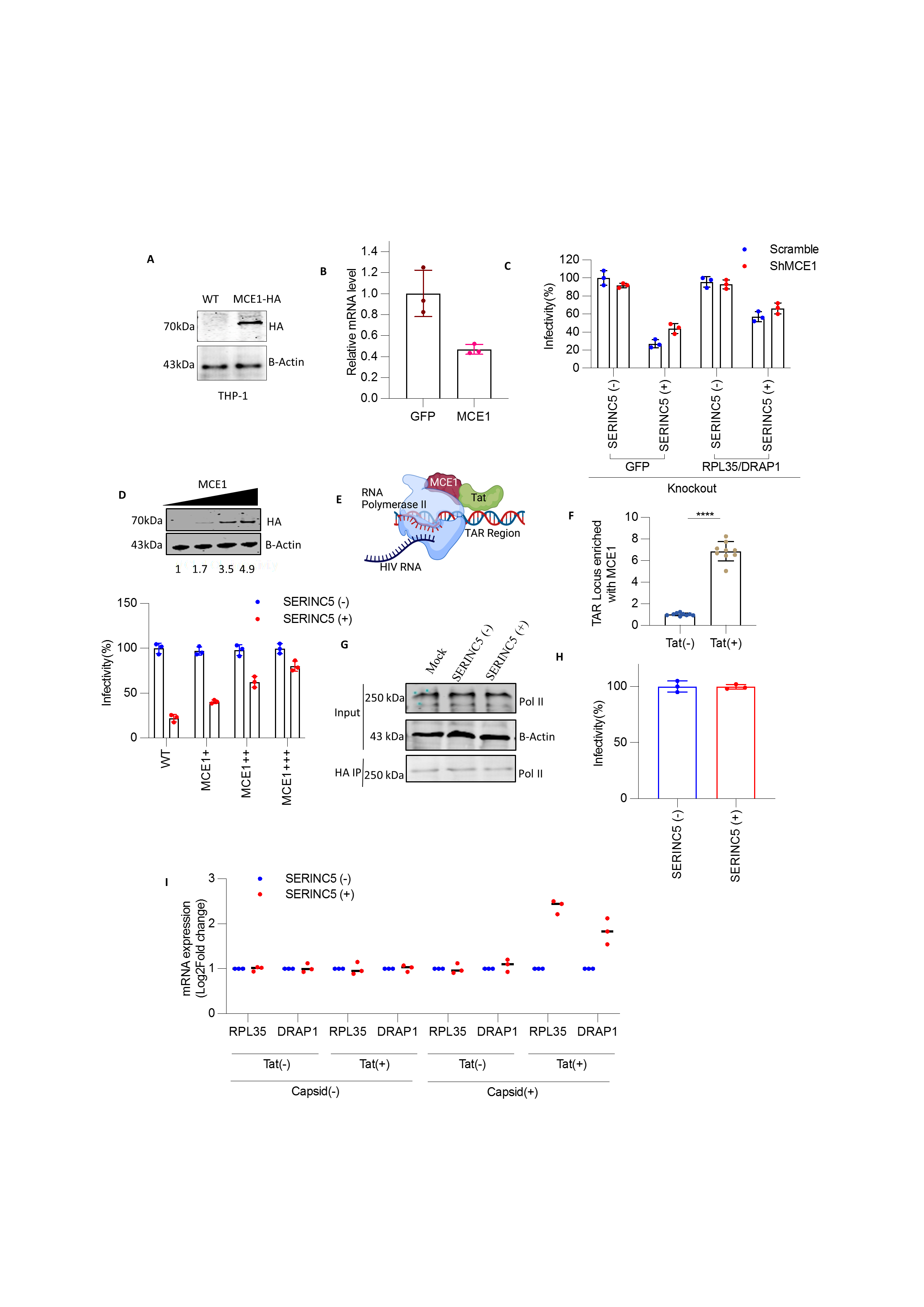

Supplement: FIG S5 [file mbio.00166-23-s0005.tif]

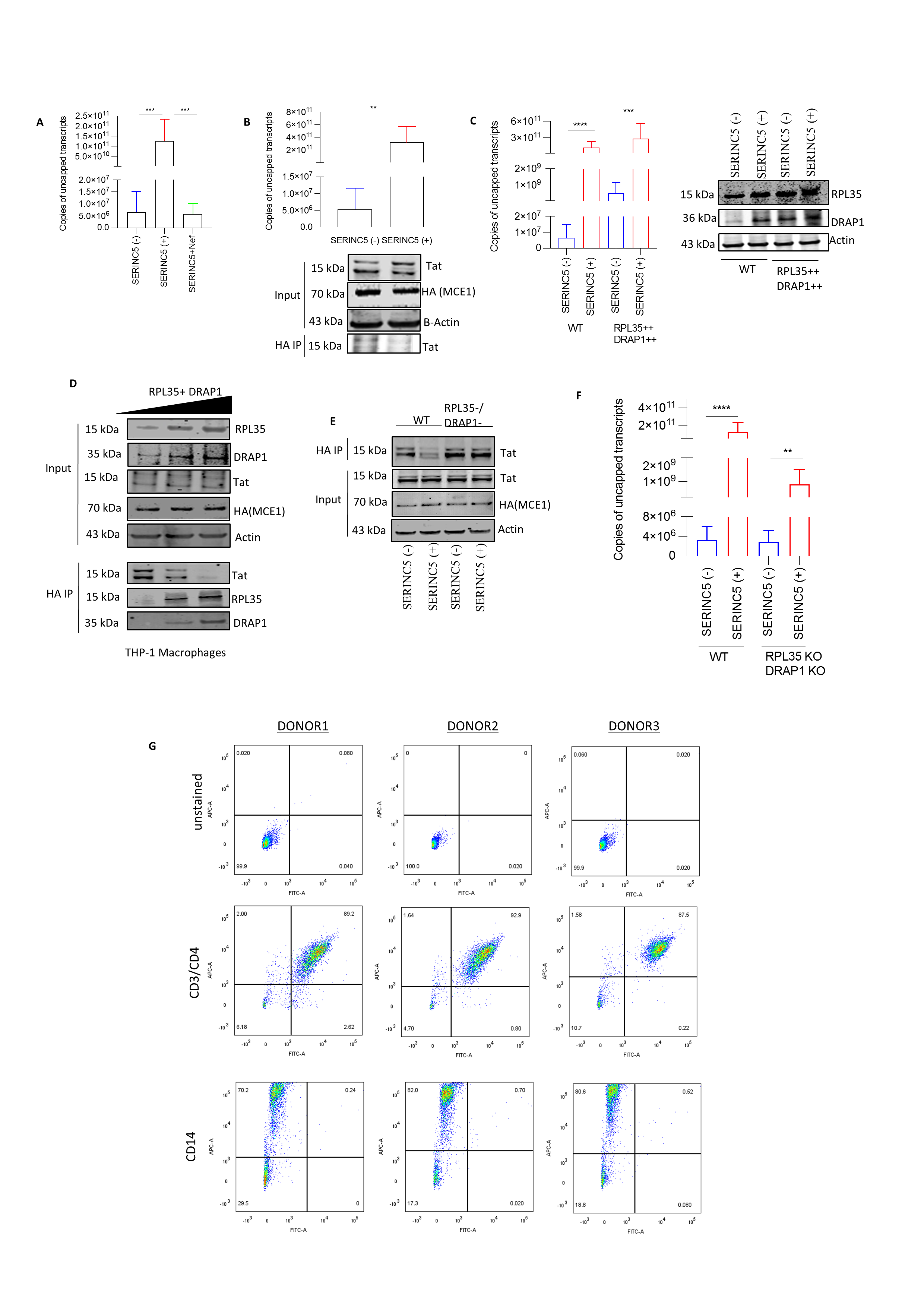

Supplement: FIG S6 [file mbio.00166-23-s0006.tif]
