# Supplementary material for: SERINC5 Mediates a Postintegration Block to HIV-1 Gene Expression in Macrophages
Source: mBio. 2023 Mar 28;14(2):e00166-23. doi: 10.1128/mbio.00166-23 (PMC10127607; doi:10.1128/mbio.00166-23)

Western blot related to Figure 2

b)

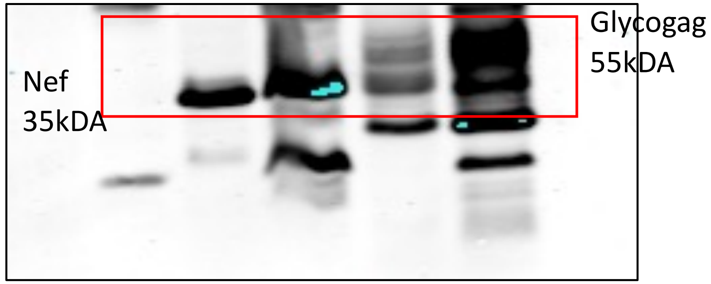

c)

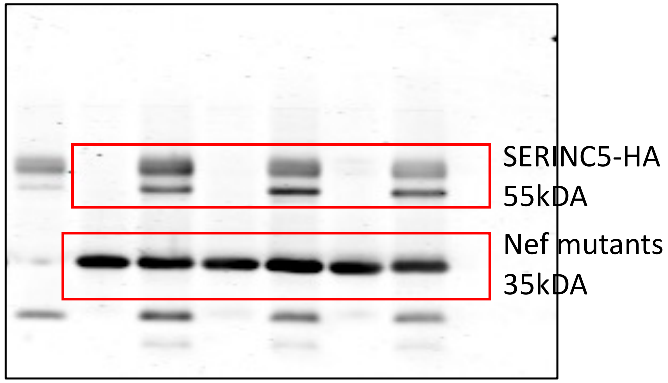

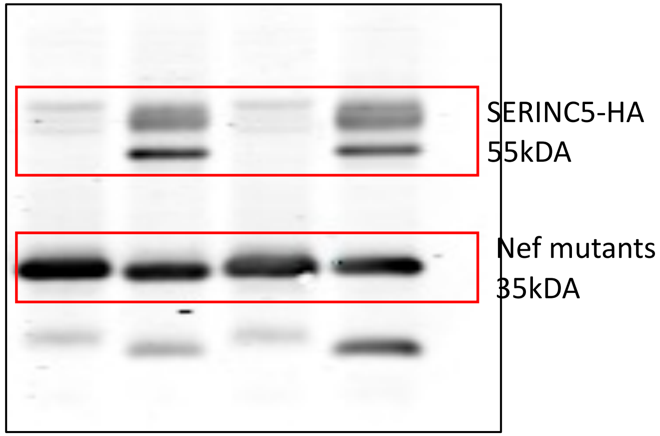

Western blot related to Figure 4

c)

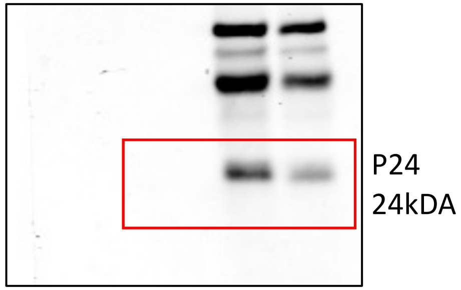

Western blot related to Figure 5

i)

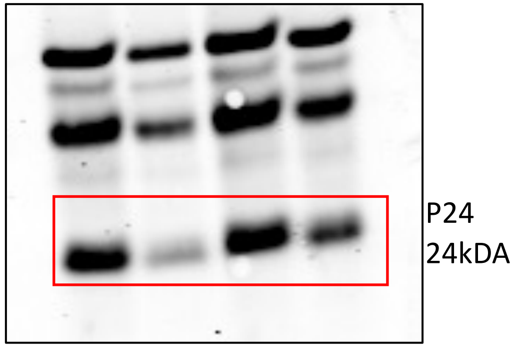

Western blot related to Figure 5

f)

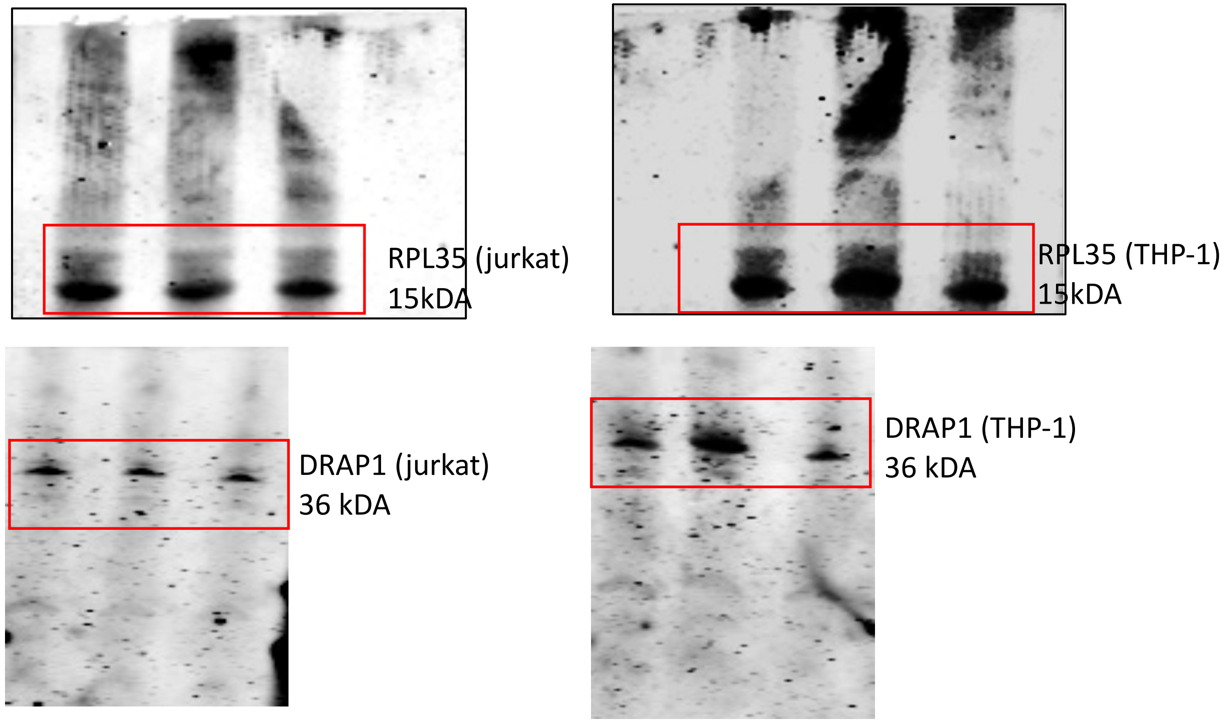

Western blot related to Figure 5

g)

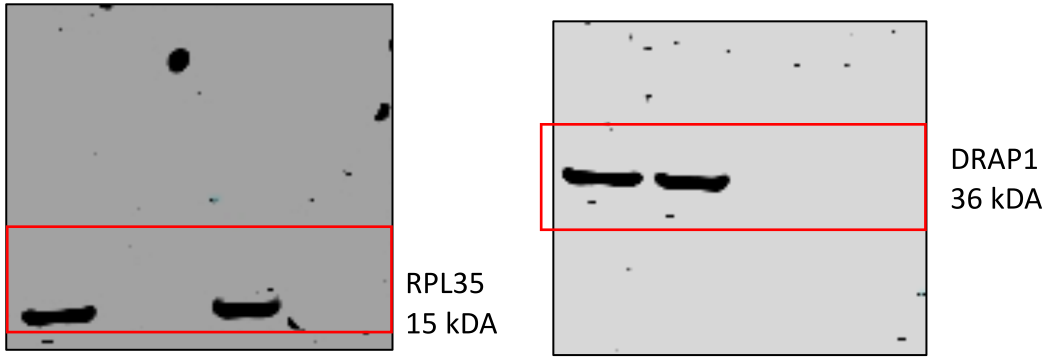

Western blot related to Figure 6

b)

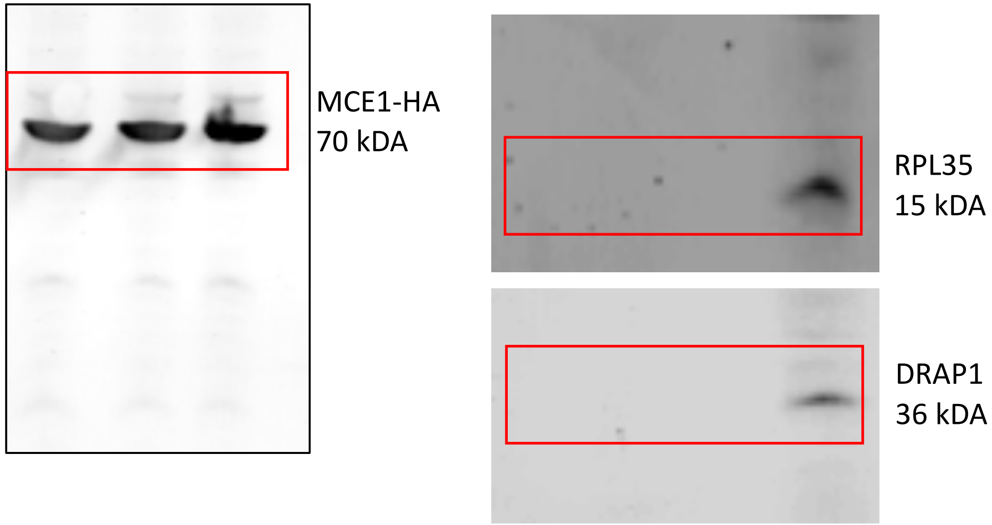

Western blot related to Figure 7

a)

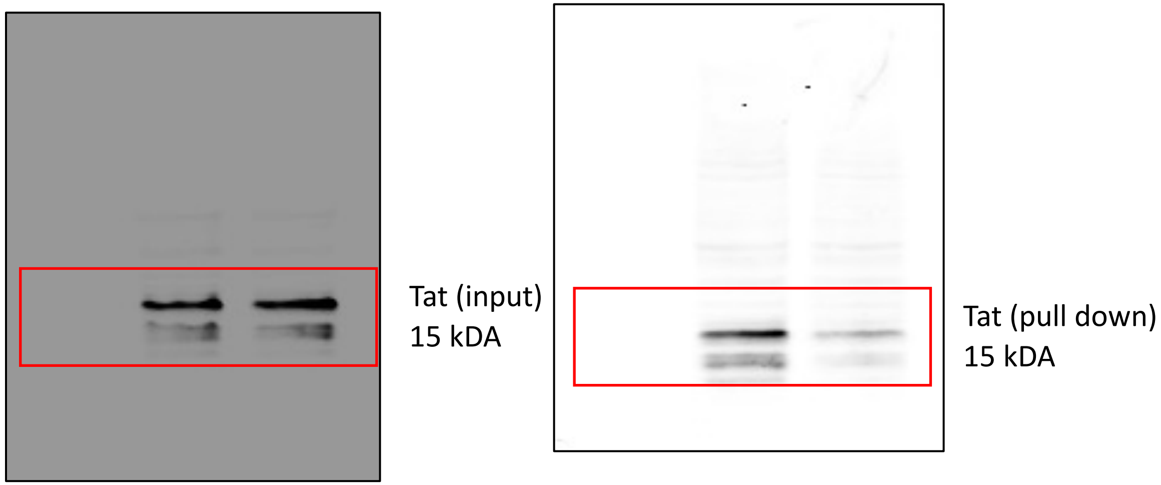

c)

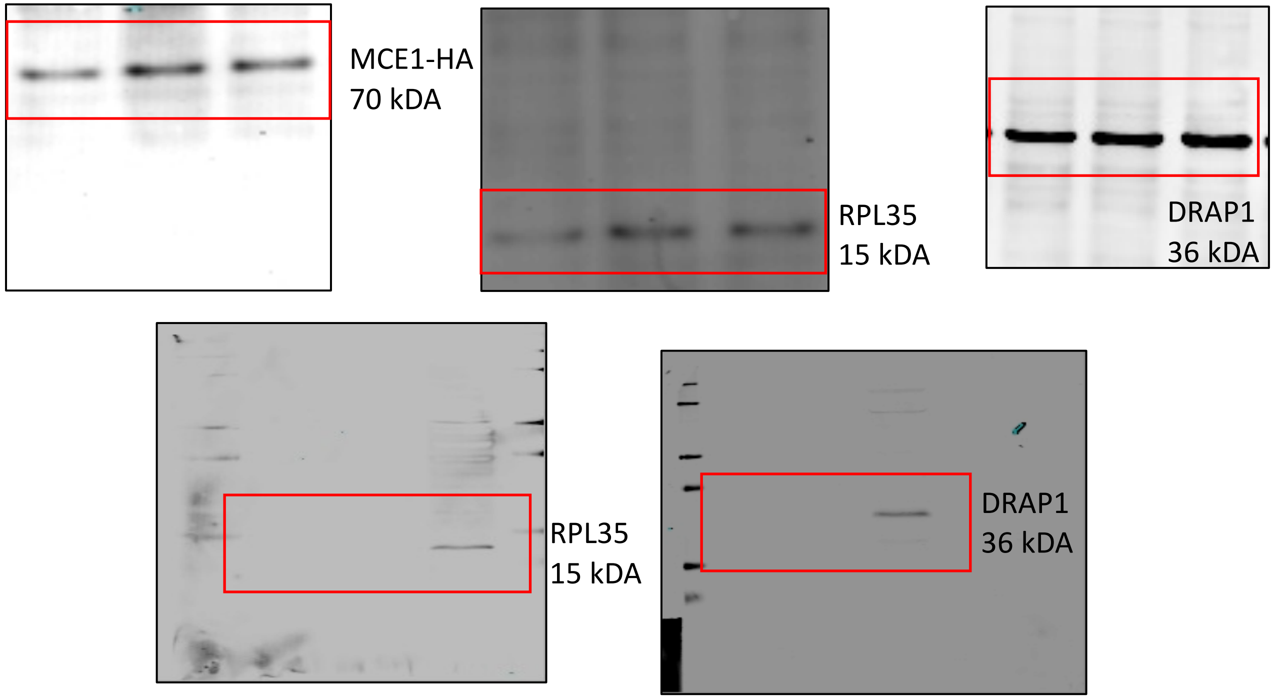

Supplement: FIG. S7 [file mbio.00166-23-s0007.pdf]
