# Supplementary material for: SERINC5 Mediates a Postintegration Block to HIV-1 Gene Expression in Macrophages
Source: mBio. 2023 Mar 28;14(2):e00166-23. doi: 10.1128/mbio.00166-23 (PMC10127607; doi:10.1128/mbio.00166-23)
Supplement: TABLE S1 [file mbio.00166-23-s0008.pdf]

SI Table I : List of plasmids and reagents used in the study

SI Table IA: List of plasmids used in the study

S.No.	Name	Purpose	Notes	Source
1.	NL4 E-R- Luc	Expression of HIV-1 laboratory strain lacking Env, Nef and Vpr ORFs	Deletion of Env, Nef, and Vpr	Prof. Massimo Pizzato NIBSC
2.	NLBN Zsgreen	Expression of HIV-1 laboratory strain lacking Env and Nef ORFs	Deletion of Env and Nef.	Prof. Massimo Pizzato
3.	pMD2.G	Expression of Vesiculostomatitis virus glycoprotein (VSV-G)		#Addgene-12259
4.	pHXB2 SRa	Expression of HIV-1 HXB2 envelope of clade B	SRa promoter driven HIV envelope	Pizzato et al., 2007 NIH AIDS Reagent Program
5.	PBJ6 SERINC5 HA	Expression of SERINC5 from a weak promoter	C-Terminal HA Tag	Rosa et al., 2015[1]
6.	PBJ5 SERINC5 HA	Expression of SERINC5 from SRa promoter	C-Terminal HA Tag	Rosa et al., 2015[1]
7.	pcDNA SERINC5 HA	Expression of SERINC5 from CMV promoter	C-Terminal HA Tag	Rosa et al., 2015[1]
8.	PBJ5 Nef Clade C HA	Expression of Nef of clade C	C-Terminal HA Tag	Pizzato et al., 2010[2]
9.	pcDNA Glycogag HA	Expression of MLV Glycogag	C-Terminal HA Tag	Pizzato et al., 2010[2]
10.	PBJ6 SERINC5 FLAG	Expression of SERINC5	C-Terminal FLAG Tag	This study
11.	PBJ5 Nef Lai HA	Expression of Nef Lai (clade B)	C-Terminal HA Tag	Pizzato et al., 2010[2]
12.	PBJ5 Nef Lai G2A HA	Expression of Nef Lai with G2A mutation	C-Terminal HA Tag	Pizzato et al., 2010[2]
13.	PBJ5 Nef Lai D123A HA	Expression of Nef Lai with D123A mutation	C-Terminal HA Tag	Pizzato et al., 2010[2]
14.	PBJ5 Nef Lai LL165AA HA	Expression of Nef Lai with LL165AA mutation	C-Terminal HA Tag	Pizzato et al., 2010[2]
15.	PBJ5 Nef Lai PP75AA HA	Expression of Nef Lai with PP75AA mutation	C-Terminal HA Tag	Pizzato et al., 2010[2]
16.	pLentiCRISPR E RPL35 TS1	SpE Cas9 and guide RNA for RPL35 encoding plasmid	gRNA target site 1	This study
17.	pLentiCRISPR E RPL35 TS2	SpE Cas9 and guide RNA for RPL35 encoding plasmid	gRNA target site 2	This study

18.	pLentiCRISPR E RPL35 TS3	SpE Cas9 and guide RNA for RPL35 encoding plasmid	gRNA target site 3	This study
19.	pLentiCRISPR E DRAP1 TS1	SpE Cas9 and guide RNA for DRAP1 encoding plasmid	gRNA target site 1	This study
20.	pLentiCRISPR E DRAP1 TS2	SpE Cas9 and guide RNA for DRAP1 encoding plasmid	gRNA target site 2	This study
21.	pLentiCRISPR E DRAP1 TS3	SpE Cas9 and guide RNA for DRAP1 encoding plasmid	gRNA target site 3	This study
22.	pScalps RPL35 HA Hygro	Lentiviral vector expressing RPL35	C-Terminal HA Tag	This study
23.	pScalps DRAP1 HA Hygro	Lentiviral vector expressing DRAP1	C-Terminal HA Tag	This study
24.	pScalps MCE1 HA Puro	Lentiviral vector expressing MCE1	C-Terminal HA Tag	This study
25.	psPax2	HIV-1 packaging plasmid		#Addgene-12260
26.	pLentiCRISPR E GFP TS1	SpE Cas9 and guide RNA for GFP encoding plasmid		Mishra et al., 2022[3]
27.	8.9 Cre	Cre expressing lentiviral construct	nlsCre inserted between MA and CA flanked by HIV-1 protease cleavage sites for processing, in 8.9 packaging plasmid	Rosa et al., 2015[1]
28.	p-lenti LoxP-Blasti-mRFP	Expression of mRFP flanked by LoxP sites		Rosa et al., 2015[1]
29.	pcDNA HIV-1 Tat	Expression of HIV-1 Tat from CMV promoter		Chande et al., 2012[4]
30.	LTR-zsgreen	Nuclear zsgreen reporter of LTR activation		Prof. Massimo Pizzato
31.	PBJ5 JR-FL	Expression of HIV-1 JR-FL envelope of clade B		Rosa et al., 2015[1] NIH AIDS Reagent program
32.	pLKO.1-puro shRNAs	Expression of shRNAs		Dharmacon
33.	pcDNA 3.1 bs(-) MCE1-HA	Expression of MCEI with CMV promoter	C-Terminal HA Tag	This study
34.	pcDNA 3.1 bs(-) RPL35	Expression of RPL35 with CMV promoter		This study
35.	pcDNA 3.1 bs(-) DRAP1	Expression of DRAP1 with CMV promoter		This study
36.	pcDNA 3.1 bs(-)	Empty vector		Rosa et al., 2015[1]<br>Life technologies
37.	pSCalps Hygro	Lentiviral vector with Hygromycin resistance		Mishra et al., 2021[5]

38.	pLentiCRISPR E	SpE Cas9 with gRNA scaffold		#Addgene-78852
39.	pSCalps zsgreen	Lentiviral vector expressing Zsgreen under Cyclophilin A promoter	3rd generation lentiviral vector	Prof. Massimo Pizzato

SI Table IB: List of Reagents used in the study

Reagent	Company	Catalog No.
Dulbecco's Modified Eagle Medium (DMEM)	Gibco, USA	12100046
RPMI 1640	Gibco, USA	11875093
Fetal Bovine Serum (FBS), Certified, Performance tested. Origin: United states	Gibco, USA	10082-147
L-Glutamax supplement	Gibco, USA	35050061
Pen-Strep (Penicillin Streptomycin)	Gibco, USA	15140-122
Hoechst 33258	Sigma Aldrich	14530-100G
OptiMEM	Gibco, USA	S18531L0102
PBS	HyClone, USA	SH30256.02
Paraformaldehyde	Sigma Aldrich	F1635-25ML
Tris(2-carboxyethyl) phosphine hydrochloride (TCEP)	Sigma Aldrich	75259
n-Dodecyl-β-D-Maltoside	Thermo Fisher	89903
2xcOmplete™, EDTA-free Protease inhibitor cocktail	Sigma Aldrich	11873580001
PVDF membrane	Immobilon-FL, Merck-Millipore	IPFL00010 R7DA8781C
Tricine	MP Biomedicals	103112 QR15009
OdysseyBlockingBuffer	Li-Cor	P/N 927-50003
Tween-20®	Sigma Aldrich	P2287-500ML
Bafilomycin A1	Santa Cruz	SC20155OA
Zidovudine (AZT)	NIH-ARP	3485
HISTOPAQUE	Sigma-Aldrich	10771
Disuccinimidyl suberate (DSS)	Thermo scientific	21655
Luciferin	Cayman Chemicals	14682-50MG
Cycloheximide	Sigma Aldrich	1810-1G
Actinomycin D	Sigma Aldrich	A1410-2MG
4-Thiouridine	Sigma Aldrich	T4509-25MG
T4 RNA Ligase	Thermo Fisher	EL0021
Dynabeads Protein G	Invitrogen	10003D
SYBR Green-I	Thermo Fisher	S-7585
TRI Reagent	Thermo Fisher	AM9738
Purified anti-HA.11 Epitope Tag antibody	BioLegend	901501
Monoclonal ANTI-FLAG® M2 antibody	Sigma Aldrich	F1804-50UG
RPL35 Polyclonal Antibody	Invitrogen	PA5-106583
DRAP1 Polyclonal Antibody	Invitrogen	PA5-52348
Beta-Actin Rabbit Monoclonal Antibody	LI-COR Biosciences	926-42210
Beta-Actin Mouse Monoclonal Antibody	LI-COR Biosciences	926-42212

IRDye 680RD Goat anti-Mouse IgG antibody	LI-COR Biosciences	925-68070
IRDye 800CW Goat anti-Rabbit IgG antibody	LI-COR Biosciences	925-32211
Anti-HIV-1 P24 antibody	NIH-ARP	1513
Mouse anti-HIV-1 Tat	NIH-ARP	7383
Rbp1 CTD Mouse Antibody	Cell Signalling Technology	2629
Human anti-CD4-APC	Miltenyi Biotec	130-113-812
Human anti-CD3-FITC	Miltenyi Biotec	130-113-690
Human anti-CD14-APC	Miltenyi Biotec	130-113-705
Recombinant anti-CD63 antibody	Abcam	Ab134045
Anti-VSV sera		Dr. Debasis Nayak

SI Table I References

1. Rosa, A. *et al.* HIV-1 Nef promotes infection by excluding SERINC5 from virion incorporation. *Nature* **526**, 212–217 (2015).
2. Pizzato, M. MLV glycosylated-gag is an infectivity factor that rescues Nef-deficient HIV-1. *Proc. Natl. Acad. Sci. U. S. A.* **107**, 9364–9369 (2010).
3. Mishra, T. *et al.* Improved loss-of-function CRISPR-Cas9 genome editing in human cells concomitant with inhibition of TGF-β signaling. *Mol. Ther. - Nucleic Acids* **28**, 202–218 (2022).
4. Chande, A. G., Baba, M. & Mukhopadhyaya, R. Short Communication: A Single Step Assay for Rapid Evaluation of Inhibitors Targeting HIV Type 1 Tat-Mediated Long Terminal Repeat Transactivation. *https://home.liebertpub.com/aid* **28**, 902–906 (2012).
5. Mishra, T. *et al.* SARS CoV-2 Nucleoprotein Enhances the Infectivity of Lentiviral Spike Particles. *Front. Cell. Infect. Microbiol.* **11**, 341 (2021).
