# Supplementary material for: SERINC5 Mediates a Postintegration Block to HIV-1 Gene Expression in Macrophages
Source: mBio. 2023 Mar 28;14(2):e00166-23. doi: 10.1128/mbio.00166-23 (PMC10127607; doi:10.1128/mbio.00166-23)
Supplement: TABLE S2 [file mbio.00166-23-s0009.pdf]

SI Table II: Sequences of shRNAs and gRNAs used

Name	Sequence
RPL35 shRNA1	CCGGGCCCGTGTTCTCACAGTTATTCTCGAGAATAACTGTGAGAACACGGGCTTTTTG
RPL35 shRNA2	CCGGGTTATTAACCAGACTCAGAAACTCGAGTTTCTGAGTCTGGTTAATAACTTTTTG
RPL35 shRNA3	CCGGCGGAAATCCATTGCCCGTGTTCTCGAGAACACGGGCAATGGATTTCCGTTTTTG
RPL35 shRNA4	CCGGGCCTAAGAAGACACGTGCCATCTCGAGATGGCACGTGTCTTCTTAGGCTTTTTG
RPL35 shRNA5	CCGGCGGGAAGAAGAAGGAGGAGCTCTCGAGAGCTCCTCCTTCTTCTTCCCGTTTTTG
NTMT1 shRNA1	CCGGGCCAAGACCTACTGGAAACAACTCGAGTTGTTTCCAGTAGGTCTTGGCTTTTTTG
NTMT1 shRNA2	CCGGGAGGTGGATATGGTCGACATACTCGAGTATGTCGACCATATCCACCTCTTTTTTG
NTMT1 shRNA3	CCGGCGGAAGTTTCTGCAGAGGTTTCTCGAGAAACCTCTGCAGAAACTTCCGTTTTTTG
NTMT1 shRNA4	CCGGCTCTTACGACGTGATCTGGATCTCGAGATCCAGATCACGTCGTAAGAGTTTTTTG
NTM1 shRNA5	CCGGGAACTACTTCTGTTGTGGGCTCTCGAGAGCCCACAACAGAAGTAGTTCTTTTTTG
DPM3 shRNA1	CCGGGCAGTGACCATGACGAAATTACTCGAGTAATTTCGTCATGGTCACTGCTTTTTG
DPM3 shRNA2	CCGGCCATGACGAAATTAGCGCAGTCTCGAGACTGCGCTAATTTCGTCATGGTTTTTG
DPM3 shRNA3	CCGGCGAAATTAGCGCAGTGGCTTTCTCGAGAAAGCCACTGCGCTAATTTCGTTTTTG
DPM3 shRNA4	CCGGCTAGCGATCCTGGGCTCCACCCTCGAGGGTGGAGCCCAGGATCGCTAGTTTTTG
DPM3 shRNA5	CCGGCCTGGGCACTGTGGGCTATCGCTCGAGCGATAGCCCACAGTGCCCAGGTTTTTG
DRAP1 shRNA1	CCGGGCACCTGATGAAGAGGACGAACTCGAGTTCGTCCTCTTCATCAGGTGCTTTTT
DRAP1 shRNA2	CCGGCTTCCTAGAGTCGCTGTTGAACTCGAGTTCAACAGCGACTCTAGGAAGTTTTT
DRAP1 shRNA3	CCGGAGGAGGATGAATCTGAGGACACTCGAGTGTCCTCAGATTCATCCTCCTTTTTT
DRAP1 shRNA4	CCGGCTTCGCCTCTACTCTGCCTTTCTCGAGAAAGGCAGAGTAGAGGCGAAGTTTTT
DRAP1 shRNA5	CCGGGACGGACGAAGAGATTGGGAACTCGAGTTCCCAATCTCTTCGTCCGTCTTTTT
MCE1 shRNA1	CCGGCTGAGAATACTGAGACCTTTACTCGAGTAAAGGTCTCAGTATTCTCAGTTTTT
MCE1 shRNA2	CCGGCGTCTGTGTGAGCGGTTTAATCTCGAGATTAAACCGCTCACACAGACGTTTTT
MCE1 shRNA3	CCGGGCCAAAGAAGTGAGCCATGAACTCGAGTTCATGGCTCACTTCTTTGGCTTTTT
MCE1 shRNA4	CCGGGCAGTGTATAGAACGAGAAATCTCGAGATTTCTCGTTCTATACACTGCTTTTT

MCE1 shRNA5	CCGGGCAGAGGCAAAGTGTAGACATCTCGAGATGTCTACACTTTGCCTCTGCTTTTT
RPL35 gRNA1	TACCCGCTGCGGAAGTACG
RPL35 gRNA2	ACTTCCTCTTTCCCTCGGAG
RPL35 gRNA3	ATCAAGGCTCGAGATCTTCG
DRAP1 gRNA1	AGGGACGCGCACCGGCC
DRAP1 gRNA2	AAGCTGTCCGGGACAGACT
DRAP1 gRNA3	AGAAGAAGAAGTACAACGCG
SERINC5 gRNA1	GCTGAGGGACTGCCGAATCC
SERINC5 gRNA2	GGCGTACCACAGCTTGTTAC
