# Supplementary material for: SERINC5 Mediates a Postintegration Block to HIV-1 Gene Expression in Macrophages
Source: mBio. 2023 Mar 28;14(2):e00166-23. doi: 10.1128/mbio.00166-23 (PMC10127607; doi:10.1128/mbio.00166-23)
Supplement: TABLE S3 [file mbio.00166-23-s0010.pdf]

SI Table III: Candidates after mass spectrometry analysis on Mascot Server

RPL35			DRAP1		
**mock treated**	**HIV-1 (SERINC5-)**	**HIV-1 (SERINC5+)**	**mock treated**	**HIV-1 (SERINC5-)**	**HIV-1 (SERINC5+)**
RL6_HUMAN 60S ribosomal protein L6	RL6_HUMAN 60S ribosomal protein L6	RL6_HUMAN 60S ribosomal protein L6	SYTL4_HUMAN Synaptotagmin-like protein 4	SYTL4_HUMAN Synaptotagmin-like protein 4	SYTL4_HUMAN Synaptotagmin-like protein 4
RL7A_HUMAN 60S ribosomal protein L7a	RL7A_HUMAN 60S ribosomal protein L7a	RL7A_HUMAN 60S ribosomal protein L7a	IGL1_HUMAN Immunoglobulin lambda-1 light chain	IGL1_HUMAN Immunoglobulin lambda-1 light chain	IGL1_HUMAN Immunoglobulin lambda-1 light chain
RL15_HUMAN 60S ribosomal protein L15	RS8_HUMAN 40S ribosomal protein S8	RL15_HUMAN 60S ribosomal protein L15	DIAP1_HUMAN Protein diaphanous homolog 1	DIAP1_HUMAN Protein diaphanous homolog 1	IGG1_HUMAN Immunoglobulin gamma-1 heavy chain
RS8_HUMAN 40S ribosomal protein S8	RL13_HUMAN 60S ribosomal protein L13	RL30_HUMAN 60S ribosomal protein L30	KDM1B_HUMAN Lysine-specific histone demethylase 1B	NEST_HUMAN Nestin	KDM1B_HUMAN Lysine-specific histone demethylase 1B
RS6_HUMAN 40S ribosomal protein S6	RL15_HUMAN 60S ribosomal protein L15	RL18_HUMAN 60S ribosomal protein L18	NEUL_HUMAN Neurolysin, mitochondrial	XP32_HUMAN Skin-specific protein 32	DIAP1_HUMAN Protein diaphanous homolog 1
RL8_HUMAN 60S ribosomal protein L8	RS6_HUMAN 40S ribosomal protein S6	H4_HUMAN Histone H4	IGG1_HUMAN Immunoglobulin gamma-1 heavy chain	DGC6L_HUMAN Protein DGCR6L	NEST_HUMAN Nestin
RL27_HUMAN 60S ribosomal protein L27	RL18_HUMAN 60S ribosomal protein L18	RS8_HUMAN 40S ribosomal protein S8	SNED1_HUMAN Sushi, nidogen and EGF-like domain-containing protein 1	KDM1B_HUMAN Lysine-specific histone demethylase 1B	MCE1_HUMAN mRNA-capping enzyme
RL13_HUMAN 60S ribosomal protein L13	RL30_HUMAN 60S ribosomal protein L30	RL27_HUMAN 60S ribosomal protein L27	IL36A_HUMAN Interleukin-36 alpha	MELT_HUMAN Ventricular zone-expressed PH domain-containing	IL36A_HUMAN Interleukin-36 alpha

				protein homolog 1	
RL30_HUMAN 60S ribosomal protein L30	RL27_HUMAN 60S ribosomal protein L27	RL13_HUMAN 60S ribosomal protein L13	NEST_HUMAN Nestin	P5CS_HUMAN Delta-1-pyrroline-5-carboxylate synthase	KV224_HUMAN Immunoglobulin kappa variable 2-24
RL14_HUMAN 60S ribosomal protein L14	RL7_HUMAN 60S ribosomal protein L7	RL4_HUMAN 60S ribosomal protein L4	P5CS_HUMAN Delta-1-pyrroline-5-carboxylate synthase	ZN878_HUMAN Zinc finger protein 878	DCD_HUMAN Dermcidin
RL3_HUMAN 60S ribosomal protein L3	IGG1_HUMAN Immunoglobulin gamma-1 heavy chain	RS6_HUMAN 40S ribosomal protein S6	ANKF1_HUMAN Ankyrin repeat and fibronectin type-III domain-containing protein 1	SNED1_HUMAN Sushi, nidogen and EGF-like domain-containing protein 1	NEUL_HUMAN Neurolysin, mitochondrial
IGG1_HUMAN Immunoglobulin gamma-1 heavy chain	RL23A_HUMAN 60S ribosomal protein L23a	HNRPU_HUMAN Heterogeneous nuclear ribonucleoprotein U	INCA1_HUMAN Protein INCA1	MED1_HUMAN Mediator of RNA polymerase II transcription subunit 1	ANKF1_HUMAN Ankyrin repeat and fibronectin type-III domain-containing protein 1
HNRPU_HUMAN Heterogeneous nuclear ribonucleoprotein U	RS24_HUMAN 40S ribosomal protein S24	H2B1C_HUMAN Histone H2B type 1-C/E/F/G/I	TACC2_HUMAN Transforming acidic coiled-coil-containing protein 2	ASAP2_HUMAN Arf-GAP with SH3 domain, ANK repeat and PH domain-containing protein 2	G3P_HUMAN Glyceraldehyde-3-phosphate dehydrogenase
RL4_HUMAN 60S ribosomal protein L4	RL8_HUMAN 60S ribosomal protein L8	IGG1_HUMAN Immunoglobulin gamma-1 heavy chain		NEUL_HUMAN Neurolysin, mitochondrial	SNED1_HUMAN Sushi, nidogen and EGF-like domain-containing protein 1
RL35_HUMAN 60S ribosomal protein L35	RL4_HUMAN 60S ribosomal protein L4	RL24_HUMAN 60S ribosomal protein L24		ALPK2_HUMAN Alpha-protein kinase 2	LIPB2_HUMAN Liprin-beta-2
RL7_HUMAN 60S ribosomal protein L7	HNRPU_HUMAN Heterogeneous nuclear ribonucleoprotein U	RL27A_HUMAN 60S ribosomal protein L27a		CP2R1_HUMAN Vitamin D 25-hydroxylase	CA194_HUMAN Protein C1orf194

RL18_HUMAN 60S ribosomal protein L18	ILF2_HUMAN Interleukin enhancer-binding factor 2	RL14_HUMAN 60S ribosomal protein L14		IGG1_HUMAN Immunoglobulin gamma-1 heavy chain	CATZ_HUMAN Cathepsin Z
RL27A_HUMAN 60S ribosomal protein L27a	RL3_HUMAN 60S ribosomal protein L3	RL3_HUMAN 60S ribosomal protein L3			CCD14_HUMAN Coiled-coil domain-containing protein 14
RL24_HUMAN 60S ribosomal protein L24	RL35_HUMAN 60S ribosomal protein L35	H15_HUMAN Histone H1.5			F110B_HUMAN Protein FAM110B
SYTL4_HUMAN Synaptotagmin-like protein 4	RS14_HUMAN 40S ribosomal protein S14	RL8_HUMAN 60S ribosomal protein L8			ARI3B_HUMAN AT-rich interactive domain-containing protein 3B
H15_HUMAN Histone H1.5	RS23_HUMAN 40S ribosomal protein S23	H3C_HUMAN Histone H3.3C			CP2R1_HUMAN Vitamin D 25-hydroxylase
RS14_HUMAN 40S ribosomal protein S14	RL14_HUMAN 60S ribosomal protein L14	RS23_HUMAN 40S ribosomal protein S23			
RS3A_HUMAN 40S ribosomal protein S3a	H15_HUMAN Histone H1.5	H2A1B_HUMAN Histone H2A type 1-B/E			
RS24_HUMAN 40S ribosomal protein S24	RS9_HUMAN 40S ribosomal protein S9	H3Y1_HUMAN Histone H3.Y			
RS26L_HUMAN Putative 40S ribosomal protein S26-like 1	RL24_HUMAN 60S ribosomal protein L24	RL7_HUMAN 60S ribosomal protein L7			
H4_HUMAN Histone H4	SYTL4_HUMAN Synaptotagmin-like protein 4	H12_HUMAN Histone H1.2			
P3C2A_HUMAN Phosphatidylinositol 4-phosphate 3-kinase C2 domain-containing	H1T_HUMAN Histone H1t	RL23A_HUMAN 60S ribosomal protein L23a			

subunit alpha					
VIME_HUMAN Vimentin	H3C_HUMAN Histone H3.3C	H1X_HUMAN Histone H1.10			
RS9_HUMAN 40S ribosomal protein S9	H3Y1_HUMAN Histone H3.Y	ACTBL_HUMAN Beta-actin-like protein 2			
KV108_HUMAN Immunoglobulin kappa variable 1-8	H4_HUMAN Histone H4	KV108_HUMAN Immunoglobulin kappa variable 1-8			
RBMX_HUMAN RNA-binding motif protein, X chromosome	RL34_HUMAN 60S ribosomal protein L34	NAMPT_HUMAN Nicotinamide phosphoribosyltransferase			
H12_HUMAN Histone H1.2	KDM1B_HUMAN Lysine-specific histone demethylase 1B	SYTL4_HUMAN Synaptotagmin-like protein 4			
RL34_HUMAN 60S ribosomal protein L34	ACTBL_HUMAN Beta-actin-like protein 2	CBLN4_HUMAN Cerebellin-4			
DIAP1_HUMAN Protein diaphanous homolog 1	RS26L_HUMAN Putative 40S ribosomal protein S26-like 1	RS2_HUMAN 40S ribosomal protein S2			
CBLN4_HUMAN Cerebellin-4	RS3_HUMAN 40S ribosomal protein S3	RL10_HUMAN 60S ribosomal protein L10			
RL23A_HUMAN 60S ribosomal protein L23a	RS2_HUMAN 40S ribosomal protein S2	P3C2A_HUMAN Phosphatidylinositol 4-phosphate 3-kinase C2 domain-containing subunit alpha			
TBA1A_HUMAN Tubulin alpha-1A chain	RBMX_HUMAN RNA-binding motif protein, X chromosome	TBA1A_HUMAN Tubulin alpha-1A chain			

RS4X_HUMAN 40S ribosomal protein S4, X isoform	CBLN4_HUMAN Cerebellin-4	RL34_HUMAN 60S ribosomal protein L34			
OPSG_HUMAN Medium-wave-sensitive opsin 1	RL27A_HUMAN 60S ribosomal protein L27a	EF1A1_HUMAN Elongation factor 1-alpha 1			
SETX_HUMAN Probable helicase senataxin	EF1A1_HUMAN Elongation factor 1-alpha 1	RL12_HUMAN 60S ribosomal protein L12			
RL35A_HUMAN 60S ribosomal protein L35a	H2B1C_HUMAN Histone H2B type 1-C/E/F/G/I	KDM1B_HUMAN Lysine-specific histone demethylase 1B			
TRPM3_HUMAN Transient receptor potential cation channel subfamily M member 3	RL22_HUMAN 60S ribosomal protein L22	C1QC_HUMAN Complement C1q subcomponent subunit C			
CABIN_HUMAN Calcineurin-binding protein cabin-1	DIAP1_HUMAN Protein diaphanous homolog 1	RL29_HUMAN 60S ribosomal protein L29			
FRY_HUMAN Protein furry homolog	TBA1A_HUMAN Tubulin alpha-1A chain	ATP9B_HUMAN Probable phospholipid-transporting ATPase IIB			
ASAP2_HUMAN Arf-GAP with SH3 domain, ANK repeat and PH domain-containing protein 2	H2A1B_HUMAN Histone H2A type 1-B/E	OASL_HUMAN 2'-5'-oligoadenylate synthase-like protein			
RS23_HUMAN 40S ribosomal protein S23	HV601_HUMAN Immunoglobulin heavy variable 6-1	RS7_HUMAN 40S ribosomal protein S7			

H1X_HUMAN Histone H1.10	RS7_HUMAN 40S ribosomal protein S7	DIAP1_HUMAN Protein diaphanous homolog 1			
CFA53_HUMAN Cilia- and flagella-associated protein 53	WNT7A_HUMAN Protein Wnt-7a	VIME_HUMAN Vimentin			
ERCC5_HUMAN DNA excision repair protein ERCC-5	RL31_HUMAN 60S ribosomal protein L31	RL35_HUMAN 60S ribosomal protein L35			
PRDM2_HUMAN PR domain zinc finger protein 2	RS16_HUMAN 40S ribosomal protein S16	RL36_HUMAN 60S ribosomal protein L36			
IGL1_HUMAN Immunoglobulin lambda-1 light chain	P3C2A_HUMAN Phosphatidylinositol 4-phosphate 3-kinase C2 domain-containing subunit alpha P	MCE1_HUMAN mRNA-capping enzyme			
ZFHX3_HUMAN Zinc finger homeobox protein 3	OPSG_HUMAN Medium-wave-sensitive opsin 1	RS3A_HUMAN 40S ribosomal protein S3a			
MY18B_HUMAN Unconventional myosin-XVIIIb OS=Homo sapiens	TRPM3_HUMAN Transient receptor potential cation channel subfamily M member 3	WNT7A_HUMAN Protein Wnt-7a			
CE152_HUMAN Centrosomal protein of 152 kDa	CFA53_HUMAN Cilia- and flagella-associated protein 53	RL36A_HUMAN 60S ribosomal protein L36a			
NEST_HUMAN Nestin	ERCC5_HUMAN DNA excision repair protein ERCC-5	BD1L1_HUMAN Biorientation of chromosomes in cell division protein 1-like 1			

H3C_HUMAN Histone H3.3C	KV108_HUMAN Immunoglobulin kappa variable 1-8	ILF3_HUMAN Interleukin enhancer-binding factor 3			
ATP9B_HUMAN Probable phospholipid-transporting ATPase IIB	SMRCD_HUMAN SWI/SNF-related matrix-associated actin-dependent regulator of chromatin subfamily A containing DEAD/H box 1	NALCN_HUMAN Sodium leak channel non-selective protein			
MED1_HUMAN Mediator of RNA polymerase II transcription subunit 1	CHM2A_HUMAN Charged multivesicular body protein 2a	UNC79_HUMAN Protein unc-79 homolog			
ILF3_HUMAN Interleukin enhancer-binding factor 3	IL25_HUMAN Interleukin-25	RL21_HUMAN 60S ribosomal protein L21			
BCLF1_HUMAN Bcl-2-associated transcription factor 1	MED1_HUMAN Mediator of RNA polymerase II transcription subunit 1	ZFHX3_HUMAN Zinc finger homeobox protein 3			
SIT1_HUMAN Signaling threshold-regulating transmembrane adapter 1	NEUL_HUMAN Neurolysin, mitochondrial	MY18B_HUMAN Unconventional myosin-XVIIIb			
RL10A_HUMAN 60S ribosomal protein L10a	HP1B3_HUMAN Heterochromatin protein 1-binding protein 3	CHM2A_HUMAN Charged multivesicular body protein 2a			
	ITPR3_HUMAN Inositol 1,4,5-trisphosphate receptor type 3	PI4KA_HUMAN Phosphatidylinositol 4-kinase alpha			
	VW5B2_HUMAN von Willebrand factor A domain-	NONO_HUMAN Non-POU domain-containing			

	containing protein 5B2	octamer-binding protein			
	RL10A_HUMAN 60S ribosomal protein L10a	I5P1_HUMAN Inositol polyphosphate-5-phosphatase A			
		CABIN_HUMAN Calcineurin-binding protein cabin-1			
		FRY_HUMAN Protein furry homolog			
		IDUA_HUMAN Alpha-L-iduronidase			
		FA76A_HUMAN Protein FAM76A			
		RS14_HUMAN 40S ribosomal protein S14			
		OPSG_HUMAN Medium-wave-sensitive opsin 1			
		HNRPC_HUMAN Heterogeneous nuclear ribonucleoproteins C1/C2			
		RL35A_HUMAN 60S ribosomal protein L35a			
		PCDA6_HUMAN Protocadherin alpha-6			
		RL10A_HUMAN 60S ribosomal protein L10a			
		AMOL2_HUMAN Angiomotin-like protein 2			

		OXND1_HUMAN Oxidoreductase NAD-binding domain-containing protein 1			
